# Nitric-Oxide-Mediated Vasodilation of Bioactive Compounds Isolated from *Hypericum revolutum* in Rat Aorta

**DOI:** 10.3390/biology10060541

**Published:** 2021-06-17

**Authors:** Hossam M. Abdallah, Noha Z. Timraz, Sabrin R. M. Ibrahim, Ali M. El-Halawany, Azizah M. Malebari, Ibrahim A. Shehata, Hany M. El-Bassossy

**Affiliations:** 1 Department of Natural Products and Alternative Medicine, Faculty of Pharmacy, King Abdulaziz University, Jeddah 21589, Saudi Arabia; noonee1408@hotmail.com (N.Z.T.); ibshehata24@hotmail.com (I.A.S.); 2 Department of Pharmacognosy, Faculty of Pharmacy, Cairo University, Cairo 11562, Egypt; ali.elhalawany@pharma.cu.edu.eg; 3 Batterjee Medical College, North Obhur, Abdullah Al-Faisal Street, Jeddah 21442, Saudi Arabia; sabrinshaur@gmail.com; 4 Department of Pharmacognosy, Faculty of Pharmacy, Assiut University, Assiut 71526, Egypt; 5 Department of Pharmaceutical Chemistry, College of Pharmacy, King Abdulaziz University, Jeddah 21589, Saudi Arabia; amelibary@kau.edu.sa; 6 Department of Pharmacology, Faculty of Pharmacy, Zagazig University, Zagazig 44519, Egypt; helbassossy@pharmacy.zu.edu.eg

**Keywords:** *Hypericum revolutum*, vasodilation, nitric oxide, xanthones, phloroglucinol

## Abstract

**Simple Summary:**

*Hypericum revolutum* (HR) is reported to produce vasodilating activity in phenylephrine-precontracted aortae, where the chloroform fraction is the most potent. Chemical investigation of this fraction yielded two new compounds, revolutin (1) and hyperevolutin C (2), along with three known metabolites, β-sitosterol (3), euxanthone (4), and 2,3,4-tirmethoxy xanthone (5). Isolated compounds 1, 2, 3, and 5 produce vasodilation activities that are dependent on endothelial nitric oxide release.

**Abstract:**

Vasodilators are an important class in the management of hypertension and related cardiovascular disorders. In this regard, the chloroform fraction of *Hypericum revolutum* (HR) has been reported to produce vasodilating activity in phenylephrine-precontracted aortae. The current work aims to identify the active metabolites in the chloroform fraction of HR and illustrate the possible mechanism of action. The vasodilation activities were investigated using the isolated artery technique. NO vascular release was assessed by utilizing the NO-sensitive fluorescent probe DAF-FM. Free radical scavenging capacity was assessed utilizing DPPH. Chemical investigation of this fraction yielded two new compounds, revolutin (1) and hyperevolutin C (2), along with three known metabolites, β-sitosterol (3), euxanthone (4), and 2,3,4-tirmethoxy xanthone (5). Compounds **1**, **2**, **3**, and **5** showed significant vasodilation activities that were blocked by either endothelial denudation or L-NAME (nitric oxide synthase inhibitor), pointing towards a role of endothelial nitric oxide in their activities. In confirmation of this role, compounds **1**–**3** showed a significant release of NO from isolated vessels, as indicated by DAF-FM. On the other hand, only compound **5** showed free radical scavenging activities, as indicated by DPPH. In conclusion, isolated compounds **1**, **2**, **3**, and **5** produce vasodilation activities that are dependent on endothelial nitric oxide release.

## 1. Introduction

Elevated blood pressure is a serious disorder that underlies other cardiovascular diseases and is a direct complication of metabolic disorders such as diabetes and metabolic syndrome. Hypertension can be due to increased heart stimulation or, most likely, increased peripheral resistance and endothelial dysfunction [1]. Endothelial dysfunction has a crucial role in the progression of hypertension by affecting vascular relaxation and constriction. The endothelium-dependent vasodilatation regulatory system controls vascular function mainly through the release of nitric oxide (NO) [2,3]. Therefore, endothelial dysfunction can lead to a significant decrease in the bioavailability of nitric oxide, causing vasodilatation impairment in affected individuals [2].

Regular medicine has many drawbacks that encourage researchers to find safer compounds for hypertension [1]. Herbal drugs are popularly dispensed for treating various ailments owing to their efficiency and comparatively moderate cost and fewer side effects. Thus, the development of effective alternative treatments is rather important in order to protect against hypertension and its complications. A literature survey has shown that many plant extracts reveal antihypertensive activity [4]. Saudi Arabia is about two million square kilometers. It covers the majority of the Arabian Peninsula. The country is renowned for many natural regions with many biologically diverse medicinal plants [5].

*Hypericum revolutum* (Vahl) is native to southeast Africa and geographically available in the Arabian mountains, especially those located in the northern and southern parts of Saudi Arabia [6]. It has been used traditionally for treating various ailments such as tuberculosis, earache, depression, diarrhea, and rheumatism, as well as burns and skin wounds due to its wound-healing property [7,8,9,10]. *H. revolutum* has been shown to possess antioxidant, antiviral, antibacterial, and anti-inflammatory activities. Hyperevolutins A and B were previously separated from the root bark and are closely related to hyperforin, previously isolated from *H. perforatum* [11]. Meanwhile, its leaves contain antifungal chromenyl ketones [12,13]. We have previously reported a significant vasodilating activity of the chloroform fraction of *H. revolutum*, produced in phenylephrine-precontracted aortae [14]. The aim of the current study is to separate and identify the biometabolites accountable for *H. revolutum* vasodilating potential. Moreover, a detailed mechanistic study of the separated compounds will be presented.

## 2. Materials and Methods

### 2.1. General Experimental Procedures

Optical rotation measurement was accomplished by a DIP-370-JASCO polarimeter. A UV–vis 1601 Shimadzu spectrophotometer was utilized for assessing UV spectra. Measuring NMR spectra was carried out using 850-INOVA BRUKER. Measuring the high-resolution mass was achieved by utilizing JEOL 102A-JMS-SX/SX and Orbitrap LTQ spectrometers. The chromatographic process was executed using RP-18 (reversed-phase) and SiO_2_ 60 (silica gel) (0.04–0.063 mm). TLC (thin-layer chromatography) was accomplished utilizing SiO_2_ 60 F_254_ TLC plates.

### 2.2. Plant Material

The aerial parts of *H. revolutum* were assembled in April 2019 from the Al-Baha governorate, KSA. No specific permission was required for the collection of this plant. Plant verification was ascertained by Dr. Emad Al-Sharif (Biology Dept., Faculty of Science and Arts, KAU). A specimen (Reg. No. HR-0438) has been deposited at the Natural Products and Alternative Medicine Department’s herbarium (Faculty of Pharmacy, *).

### 2.3. Plant Material Extraction

Dried powdered aerial parts (1 Kg) were extracted at room temperature with 5 L methanol (four times), utilizing Ultraturrax, until exhausted. The under-vacuum concentration of the total extract produced a brown residue (40 g). The residue was mixed with 200 ml of water, partitioned with 500 ml chloroform (4 times), and vaporized to furnish Fraction I (10 g).

### 2.4. Separation of Major Metabolites

A 10 g chloroformic fraction was submitted to vacuum liquid chromatography (VLC) using silica gel (SiO2) (10 × 15 cm), utilizing an n-hexane/ethyl acetate (EtOAc) gradient. One hundred milliliter fractions were gathered and subjected to SiO_2_ TLC plates [solvent systems: n-hexane/EtOAc, 95:5 (S1) or 90:10 (S2)] and similar fractions were combined to furnish four main subfractions (I to IV). Subfraction I (50 mg, 13–15, 97:3; S3) included two main spots that yielded red color upon spraying with *p*-anisaldehyde:H_2_SO_4_ reagent, which was purified by SiO_2_ column chromatography (CC) using an S3-solvent system. The related fractions (20 mL each) were assembled on the basis of TLC (S1). The fractions (5–15, 20 mL each; 20–30, 20 mL each) each comprised one main spot that was additionally repurified on RP-18 CC (MeOH/water, 80:20) to provide **1** (4 mg) and **2** (20 mg). SiO2 CC of Subfraction II (50 mg, n-hexane/EtOAc 93:7; S4) yielded **3** (15 mg) and **4** (12 mg). Subfraction III (24–26, S2), having one main spot, was submitted to SiO2 CC (2 × 40 cm, S4) to furnish **5** (7 mg).

### 2.5. Biological Evaluation

#### 2.5.1. Chemicals and Drugs

ACh (acetylcholine), PE (phenyl ephedrine), DMSO (dimethyl sulfoxide), and DPPH (2,2-diphenyl-1-picrylhydrazyl) were acquired from Sigma–Aldrich (Dorset, UK); DAF-FM (4-amino-5-methylamino-2’,7’-difluorofluorescein di-acetate) was acquired from Molecular Probes (New York, NY, USA). Deionized-Ultrapure H_2_O was utilized as solvent except for DPPH and natural metabolites, where DMSO (conc. not exceeding 0.1%) was utilized.

#### 2.5.2. Animals

Seven-week-old male Wistar rats (180–200 g) were used (King Fahd Medical Research Center, KAU, KSA). The animals were housed with access to standard rodent pellets and purified water in clear polypropylene cages (4 rats each). Constant housing conditions were applied, including alternating 12 h light and dark, 22 ± 3 °C temperature, sufficient ventilation, and 50–60% relative humidity. The research ethics committee of King Abdulaziz University approved the study (approval number 126-1439). The study was carried out according to the Saudi Arabia Research Bioethics Guidelines and Regulations, which are in accordance with the Animals in Research: Reporting In Vivo Experiments (ARRIVE) guidelines for research involving animals [15]. The animals were executed by decapitation using a rodent guillotine administered by qualified personnel in the animal housing. The method is acceptable to induce a rapid loss of consciousness, according to the AVMA Guidelines for the Euthanasia of Animals: 2020 Edition (section M3.7) [16], and the descending thoracic aorta was precisely removed and washed from connective tissues and fats.

#### 2.5.3. Evaluating the Chloroform Fraction and Isolated Metabolites’ Direct Relaxant Effect

Vasodilating capacities were assessed using the isolated artery method, as formerly reported [17,18]. Briefly, the aorta was removed, cleansed of any connective tissue and fats, and sliced into rings (3 mm). Each ring was hung in Krebs Henseleit buffer channels (4.8 mM KCl, 118 mM NaCl, 1.2 mM MgSO_4_, 1.2 mM KH_2_PO_4_, 2.5 mM CaCl_2_, 11.1 mM glucose, and 25 mM NaHCO_3_) at 37 °C, with continuous aeration with gas (5% CO_2_ and 95% O_2_). Every 30 min, the channel buffer solution was exchanged. Quantification of the aortic tension was accomplished using an isometric force transducer, and the results were presented through a PowerLab data interface module linked to a PC running Chart software v8 (ADI Instruments).

The aortic rings were set aside for 30 min for equilibration at a 1500 mg ± 50 resting tension. Initial aorta contraction and relaxation were then carried out by the addition of PE, followed by ACh (both at 10 μM). After the tension was reverted to the rest state, accumulative concentrations of 1–10 μg/mL and 1–10 μM for the chloroform fraction or the pure metabolites, respectively, were added to the organ bath precontracted (PE, 10 μM)-isolated aortae. Tension reduction was estimated as a measurement of vasodilating actions. In other sets of experiments for investigating the role of the endothelium in the vasodilating effect, it was mechanically made bare. Additionally, L-NAME (100 μM) was added in the organ bath 15 min before adding the isolated metabolites or chloroform fraction to explore the role of nitric oxide in the vasodilating influence on different sets of experiments.

#### 2.5.4. Examining the Effect of Chloroform Fraction and Isolated Metabolites on Nitric Oxide (NO) Production

The separated aorta intracellular induction of NO production by the tested metabolites and the chloroform fraction was examined employing the DAF-FM fluorescence probe, as similarly outlined in our former work [19]. Similar to the earlier technique, the thoracic aorta was removed, the fats were washed off, and it was sliced into approximately 6 mm pieces. Every piece was put in a 96-well black plate separate well, maintained in dim light, having made a 2.5 µM DAF-FM/KHB mixture (37 °C) immediately before starting the procedure; 100 µL were accurately drawn and transported to the wells’ neighboring column; after that, ACh (10 µM) was included in one of the aortic segments, and the chloroform fraction (10 µg/mL) or separated metabolites (10 μM) was put in the other wells after 3 min. Again, after 3 min, 100 µL were taken from the wells with the aortae and transmitted to the nearby well columns. For the blank, a row without an aorta was retained for each ACh concentration, which was handled identically. The withdrawn volumes’ fluorescence intensity (and not the column including the aortae) was then assessed at λem = 515 nm and λex = 485 nm using a SpectraMax^®^ M3 Monochromator plate reader.

#### 2.5.5. Studying the ROS Scavenging Potential of the Isolated Metabolites and the Chloroform Fraction

ROS scavenging potential was assessed, as formerly stated in previous work from our laboratories [20]. In a 96-well clear plate, the pure metabolites (1–10 µM) or Fraction I (1–10 µg/mL) in MeOH was added to a DPPH (240 µM) solution in MeOH/tris (1:1 *v*/*v*). For the control (C), MeOH was utilized instead of the fraction or metabolites. DPPH was directly prepared before adding to the plate. The absorbance was estimated every minute at 520 nm for 10 min using a SpectraMax^®^ M3-Monochromator plate reader.

#### 2.5.6. Statistical Analysis

Experimental values are depicted as the mean ± standard error of the mean (SEM). For statistical analysis, one- or two-way ANOVA (analysis of variance) was applied, as designated in the figure legends, succeeded by Dunnett’s posthoc test utilizing GraphPad Instat software version 5. If *p* < 0.05, the differences were recognized as significant.

## 3. Results

### 3.1. Identification of Isolated Compounds

Chemical examination of *H. revolutum* aerial parts’ CHCl_3_ fraction gave rise to the separation of two new constituents (**1** and **2**) and three known ones (**3**–**5**) (Figure 1 and Figures S1–S22, Tables S1–S3). Their structures were characterized using one- and two-dimensional nuclear magnetic resonance (1D and 2D NMR) and mass (MS) analyses, as well as a comparison with the literature. The known metabolites were euxanthone (1,7-dihydroxyxanthone) (**4**) [21], β-sitosterol (**3**) [22], and 2,3,4-tirmethoxy xanthone (**5**) [23].

### 3.2. Compound 1

**Figure 2:**
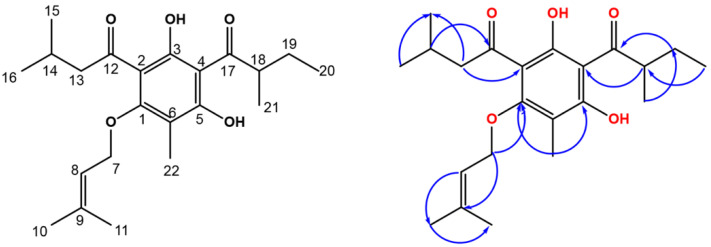
Chemical structure of compound **1** and some HMBC correlations.

Compound **1** was separated as a yellow amorphous powder, having a C_22_H_32_O_5_ molecular formula, as assigned by HRESIMS (Figure S1), that possessed a pseudomolecular peak at m/z 377.2335 [M+H] ^+^. The ^13^C and ^1^H NMR data illustrated that **1** had a phloroglucinol framework (Table 1, Figure 2, Figures S2 and S3) [24,25,26]. The phloroglucinol skeleton’s existence was endorsed from the noticed three oxygen-linked aromatic carbons, C-5 (δ_C_ 162.5), C-1 (δ_C_ 162.4), and C-3 (δ_C_ 149.9), and the three quaternary aromatic carbons, C-4 (δ_C_ 104.3), C-2 (δ_C_ 104.5), and C-6 (δ_C_ 104.8) [26,27,28], in ^13^C spectrum. The heteronuclear single quantum coherence (HSQC) (Figure S4) and ^13^C NMR featured 22 carbons: 3 methylenes, 7 methyls, 3 methines, and 9 quaternaries, including 2 carbonyls for ketone C-12 (δ_C_ 205.7) and C-17 (δ_C_ 210.3). The noticed signals for methylene (H-19, δ_H_ 1.41 and 1.84), a multiplet methine (H-18, δ_H_ 3.73), a secondary methyl (H-21, δ_H_ 1.16), and a primary methyl (H-20, δ_H_ 0.91), having HSQC correlations to the carbons at δ_C_ 26.9, 46.0, 16.7, and 12.0, respectively, represented a 2-methylbutyryl moiety [29]. The heteronuclear multiple bond correlation (HMBC) correlations (Figure S5) of H-18 to C-19, C-17, C-21, and C-20, H-19 to C-18, C-17, C-21, and C-20, H-20 to C-19 and C-18, and H-21 to C-19, C-18, and C-17 connect this moiety to phloroglucinol at C-4. The ^13^C and ^1^H spectra disclosed signals for a 3-methylbutyryl moiety at δ_C_ 52.9 (C-13)/δ_H_ 2.94 (H-13), 25.3 (C-14)/1.65 (H-14), 22.8 (C-15, 16)/0.97 (H-15, 16), and δ_C_ 205.7 (C-12). This was ensured by the cross-peaks of H-16 and H-15/C-14 and C-13 and H-14 and H-13/C-16, and C-12, and C-15 in HMBC. In HMBC, the cross-peak of H-13 to C-2 set up the location of this chain at C-2 of the phloroglucinol skeleton (Figure 2). The ^13^C and ^1^H NMR data revealed signals for an oxymethylene [δ_C_ 65.3 (C-7)/δ_H_ 4.52 (H-7)], a tri-substituted olefinic bond [δ_C_ 119.2 (C-8)/δ_H_ 5.45 (H-8)], and two methyls [δ_C_ 18.3 (C-11)/δ_H_ 1.73 (H-11) and δ_C_ 25.8 (C-10)/δ_H_ 1.79 (H-10)], denoting the presence of a 3-methylbut-2-enoxy moiety in **1** that was ascertained by HMBC peaks [30,31]. Its connection to C-1 was affirmed by the cross peak of H-7 to C-1 in HMBC. Moreover, a signal for a methyl (δ_H_ 2.02, H-22), having an HSQC cross-peak to a carbon at δ_C_ 7.3 and HMBC cross-peaks to C-6, C-1, and C-5, characteristic for a C-6 aromatic methyl. Finally, **1** was assigned as phloroglucinol derivative and named revolutin.

### 3.3. Compound 2

Compound **2** was a yellow amorphous solid, having two pseudo-molecular ion peaks at m/z 537.2821 (C_30_H_45_^37^ClO_6_, [M+H]^+^) and 535.2816 (C_30_H_45_^35^ClO_6_, [M+H]^+^) in a 1:3 ratio in HR-ESI-MS (Figure S6), suggesting that **2** has a chlorine atom [32]. This formula requires nine unsaturation degrees. The ^1^H spectrum revealed signals for four quaternary methyls [H-14 (δ_H_ 1.24), H-12 (δ_H_ 1.26), H-13 (δ_H_ 1.28), and H-11 (δ_H_ 1.38)], two γ,γ-dimethyl allyls, two secondary methyls [H-18 (δ_H_ 1.09) and H-17 (δ_H_ 1.10)], a methine (H-3), an oxymethine (H-19), and three methylenes (H-4, -5, and -25) (Table 2).

**Figure 3:**
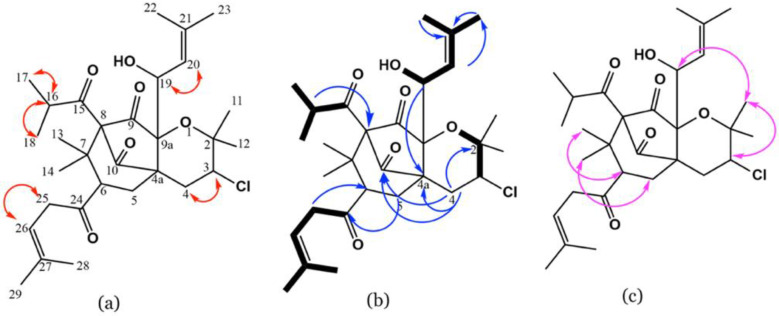
Important COSY (**a**), HMBC (**b**), and NOESY (**c**) correlations of **2**.

Its HSQC and ^13^C (Figures S8 and S9) spectra exhibited 3 methylenes, 10 methyls, 6 methines (from which there were one oxymethine and two olefinics), and 11 quaternaries comprising 4 carbonyls. ^1^H-^1^H correlated spectroscopy (COSY) of **2** (Figure S11) displayed cross-peaks of H-17/H-16 and H-18, H-19/H-20, H-3/H-4, and H-26/H-25 that confirmed the partial substructures demonstrated in bold lines (Figure 3a). The HMBC relations (Figure S10) of H-25 to C-27, C-24, and C-6 secured the connection of C-6 and C-26, C-24, and C-25 and affirmed the linking of the 1-oxo-4-methyl-pent-3-eneyl moiety at C-6 (Figure 3b). Further, the linkage of γ,γ-dimethyl allyl at C-25 was assured by the relations of H-26 to H-29 and H-28. Furthermore, the cross-peaks between H-5/C-7 and C-24 assured the linkage between C-5, C-6, and C-7. The cross-peaks of H-16/C-18, C-15 and C-17, H-14 and H-13/C-8 and C-6, and H-16/C-8, besides relation H-6/C-8, assured the connection between C-8/C-7 and C-7/C-6 and secured the location of the 1-oxo-2-methyl propyl moiety at C-8. The correlations of H-4/C-4a, C-9a, C-10, C-5, and C-2, and H-12 and H-11/C-2 in HMBC proved the 3,4-dihydropyran moiety and the connection between C-10 and C-4a. This evidence revealed the fusion of the pyran ring at C-4a and C-9a. The linkage of the γ,γ-dimethyl ally group to C-9a via a C-19 oxymethine was assured by the HMBC cross-peaks of H-20/C-23 and C-22, H-23, H-22, and H-19/C-21, and H-19/C-4a and C-9. The downfield shift of C-3/HC-3 established the existence of a chloride at C-3. Further, the nuclear Overhauser effect spectroscopy (NOESY) spectrum of **2** (Figure S12) displayed prominent cross-peaks between H-12/Hβ-4, H-3 and H-19/H-11, H-14/Hβ-5, and H-13/H-6, suggesting the existence of H-3, H-11, H-13, H-6, H-16, and H-19 on the same face of the molecule (Figure 3c). Therefore, the structure of **2** was elucidated as represented and named hyperevolutin C. The nomenclature of **2** was given based on its structural similarity to hyperevolutins A and B, which were previously separated from *H. perforatum* [11].

### 3.4. Chloroform Fraction Vasodilating Activity

**Figure 4:**
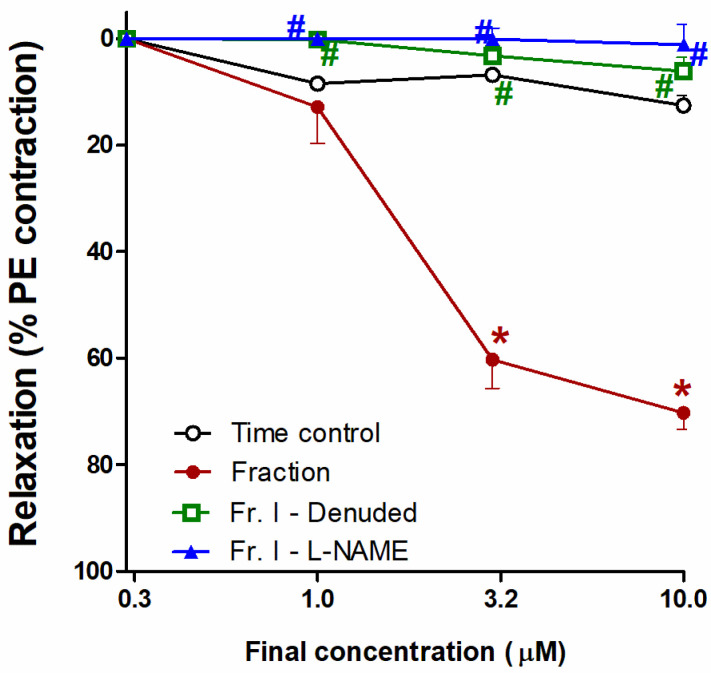
Effect of the *H. revolutum* chloroform fraction on PE-precontracted separated intact or denuded aorta or aorta preincubated with a nitric oxide synthase inhibitor (L-NAME) compared with time control. Results are introduced as the mean ± standard error of the mean (SEM) (*n* = 8). * Significantly varies from the corresponding time control values (*p* < 0.05); # Significantly varies from the corresponding chloroform fraction (*p* < 0.05) by two-way repeated-measures ANOVA and the Newmans–Keuls posthoc test.

The chloroform fraction exhibited reductions in tension and consequent concentration-dependent vasodilation of PE (10 μM)-precontracted separated aortae that approached statistical significance compared with time control (at conc. 3 and 10 µg/mL, both at *p* < 0.05). Removal of the endothelial layer (aorta denudation) prevented the chloroform fraction vasodilating effect, as obvious from the considerable prohibition (at conc. 1, 3, and 10 µg/mL, all at *p* < 0.05). Additionally, L-NAME (1 mM) blocked the vasodilating effect of the chloroform fraction, as manifest from the remarkable inhibition (at conc. 1, 3, and 10 µg/mL of the choroform fraction, all at *p* < 0.05) (Figure 4).

### 3.5. Vasodilating Activity of the Isolated Compounds

**Figure 5:**
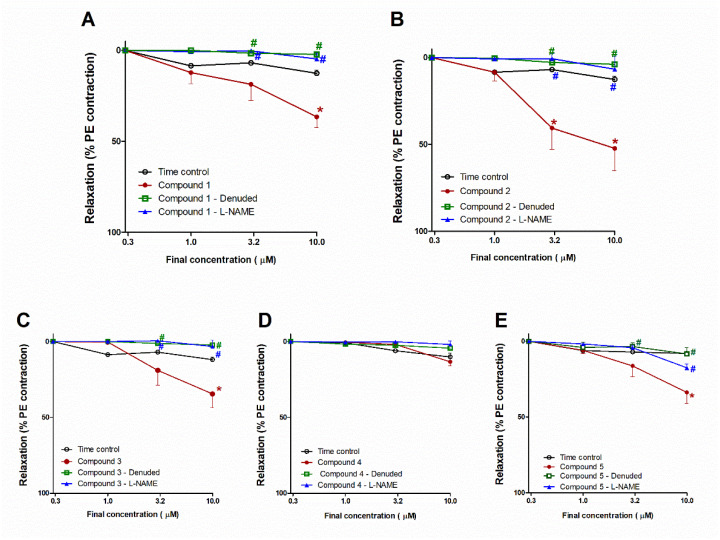
Influence of compounds **1**–**5** on PE–precontracted separated intact or denuded aorta or aorta preincubated with a nitric oxide synthase inhibitor (L-NAME) compared with time control (**A**–**E**). Results are introduced as mean ± SEM (*n* = 8). * Significantly varies from the corresponding time control values (*p* < 0.05). # Significantly varies from the corresponding values of compound **1** (*p* < 0.05) by two-way ANOVA and Newmans–Keuls posthoc test.

Figure 5A reveals that compound **1** caused the concentration-dependent vasodilation of PE (10 μM)–precontracted separated aortae, as apparent from the notable repression of vasodilation (at conc. 10 µM, *p* < 0.05), whilst L-NAME or endothelial denudation effectively hindered the vasodilation potential of compound **1**, as indicated by the remarkable inhibition (both at conc. 3 and 10 µM, all at *p* < 0.05; Figure 5A). Likewise, compound **2** caused concentration-dependent vasodilation (at conc. 3 and 10 µM, both *p* < 0.05); however, L-NAME or endothelial denudation prevented the activity (both at conc. 3 and 10 µM, all at *p* < 0.05; Figure 5B).

**Figure 6:**
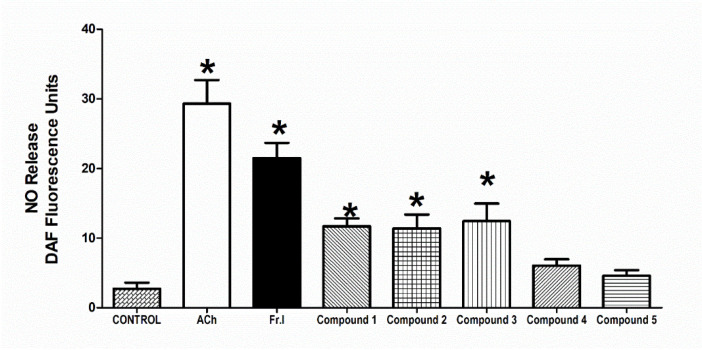
Influence of *H. revolutum*, 10 µg/mL Fraction I, and compounds **1**–**5** (conc. 10 µM) on vascular NO production comparable to 10 µM Ach. * Significantly varies from the control value (*p* < 0.05) by one way-ANOVA and Newmans–Keuls posthoc test.

The compound **3** addition resulted in concentration-dependent vasodilation of PE (10 μM)–precontracted separated aortae (Figure 5C). At a concentration of 10 µM, the vasodilating effect of compound **3** attained a statistically significant level (*p* < 0.05) compared with time control. This effect was entirely blocked by L-NAME or endothelial denudation, as apparent from the notable prohibition (both at conc. 3 and 10 µM, all at *p* < 0.05; Figure 6A). Nevertheless, compound **4** did not possess any notable vasodilation of PE (10 μM)–precontracted separated aortae. Likewise, L-NAME or endothelial denudation had no significant influences (Figure 5D).

Figure 5E demonstrated that the compound **5** addition gave rise to a decline in tension and, consequently, concentration-dependent vasodilation of PE–precontracted separated aortae. This effect at 10 µM attained a statistically significant (*p* < 0.05) level. Moreover, this effect was inhibited by endothelial denudation (at conc. 10 µM) or L-NAME (at both conc.3 and 10 µM, all at *p* < 0.05; Figure 5E).

### 3.6. Effect on Vascular NO-Production

The addition of 10 µM Ach at 37 °C to the aortic rings caused a marked NO production (*p* < 0.05) in comparison to control that was quantified and detected using 2.5 µM DAF-FM reagent. The chloroform fraction addition (conc. 10 µg/mL) brought about a similar increase in NO production, as evident by a notable increase in the fluorescence of DAF-FM compared with the control values (*p* < 0.05). The isolated metabolites **1**–**3** provoked NO production that attained a statistically noteworthy level (all at *p* < 0.05) at a 10 µM concentration (Figure 6).

### 3.7. Free Radical Scavenging (FRS) Capacities

**Figure 7:**
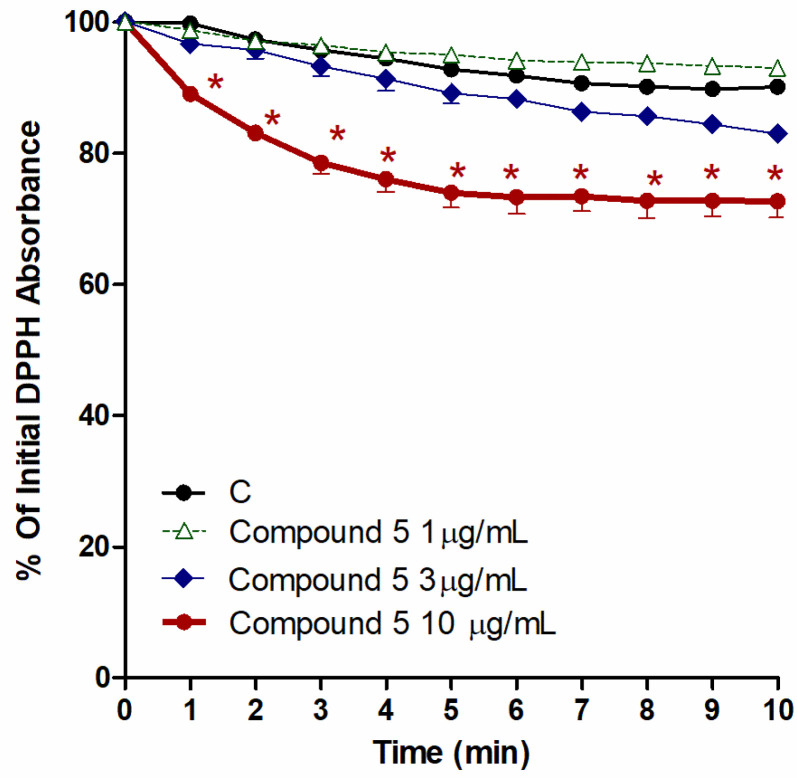
Influence of the CHCl_3_ fraction and isolated metabolite **5** on the production of ROS, as inducted by 240 µM DPPH. Control (C) is a reaction mixture with DPPH only. Results are displayed as mean ± SEM (*n* = 3). * *p* < 0.05 when compared to each corresponding control.

In the 10 min reaction results between 240 µM DPPH, the chloroform fraction, and compounds **1**–**5** (conc. 1, 3, and 10 µM), only **5** exhibited remarkable FRS activity. The other compounds, **1**–**4**, did not have any significant FRS activities (data not shown). Figure 7 revealed that **5** (conc. 10 µM) possessed DPPH free radical scavenging activity that was translated into an antioxidant potential, which is clear from the noticeable variations from control, beginning from the 1st minute until the 10th minute (*p* < 0.05).

## 4. Discussion

Natural products can exert vasodilation through either the endothelium (NO generation) or by acting on smooth muscle (Ca^+^ and K^+^ channels) [33]. The current work represents the first evaluation of the bioactive compounds from *H. revolutum* that are responsible for vasodilation activities; we also investigate the mechanism of action. A previous report from our laboratory [14] proved that the total methanol extract of *H. revolutum* gave rise to concentration-dependent vasodilation of phenylephrine–precontracted isolated aortae. The bio-guided fractions indicated that the chloroform fraction is accountable for the noticed total extract vasodilation potential. In this regard, similar vasodilating activities have been reported for other plant extracts; the methanol extract of *Garcina mangostana* as well as *Mentha longifolia* were reported to produce a direct vasorelaxant effect in phenylephrine-induced vasoconstriction and in an experimental model of angina, respectively [34,35]. Moreover, different phytoconstituents are known for their vasodilation activity. Phenolic compounds are the most important class of vasodilators. For example, flavone can exert vasodilation by acting on the Ca^+^ channel and NO generation [33].

Chromatographic examination of the chloroform fraction gave rise to the separation of five active compounds that worked collaboratively but through different mechanisms to prompt NO-dependent vasodilatation. The isolated compounds were identified as revolutin (**1**) and hyperevolutin C (**2**), β-sitosterol (**3**), euxanthone (1, 7-dihydroxyxanthone, **4**), and 2,3,4-tirmethoxy xanthone (**5**).

**Figure 8:**
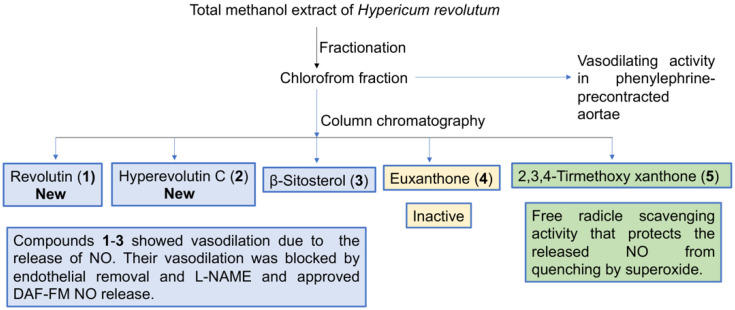
Diagrammatic sketch summarizing the study design.

Compounds **1**, **2**, and **3** show significant vasodilation activities that were blocked by endothelial denudation (Figure 8). This points to the key role of the endothelium in mediating their vasodilating activities. In addition, L-NAME (NO synthase inhibitor) completely blocked the mentioned compounds’ activities, suggesting endothelial nitric oxide stimulation as a major mechanism of activity. It is well established that the endothelium has a crucial role in controlling arterial tone via the release of the key vasorelaxant molecule NO [36]. In order to confirm that endothelial NO stimulation is the main vasodilating mechanism of compounds **1**, **2**, and **3**, the endothelial release of NO upon compound addition was measured by the NO-sensitive fluorescent probe, DAF-FM. The current study confirms the release of NO from the vascular endothelium upon the addition of compounds **1**, **2**, and **3**.

Revolutin (**1**) belongs to the phloroglucinol group of compounds, which are known for their ability to improve NO generation. Previous reports have reported its ability to increase NO levels, leading to a decrease in blood pressure in vivo [37]. Meanwhile, hyperevolutin C (**2**) is a novel terpenoid structure closely related to garcinielliptone G, which was previously isolated from *Garcinia subelliptica* [38] but not previously tested for its biological effects. β-Sitosterol (**3**) has previously exhibited hepatoprotective and cardioprotective effects in CdCl_2_-induced hypertensive rats [39]. Euxanthone (**4**) was previously investigated and showed a pronounced vasodilator effect through the release of endothelial factors such as NO and COX-derived factors. Additionally, it provoked the prohibition of a Ca^+2^-sensitive mechanism initiated by protein kinase C instead of repression of a contraction-dependent release of the intracellular Ca^+^ stores or prohibiting voltage-operated Ca^+^ channels [40].

**Figure 9:**
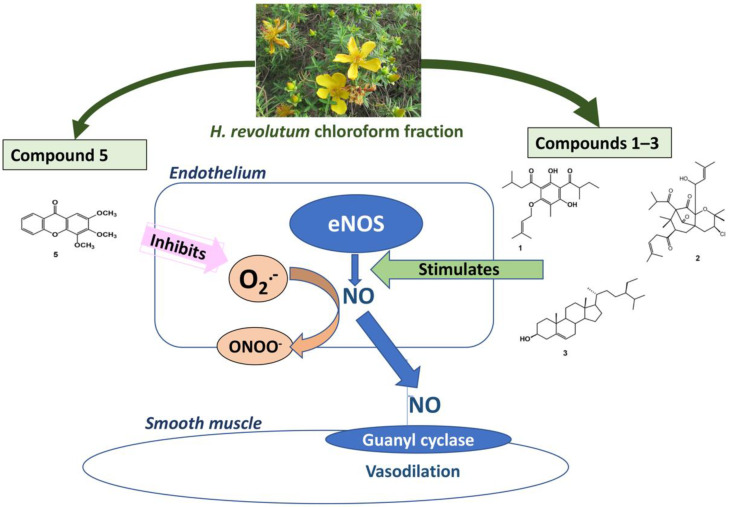
Diagrammatic sketch summarizing the proposed pharmacological mechanism.

Free radical scavenging (FRS) activity is another important way to preserve the released NO from quenching by superoxides and subsequent conversion into nitrites or nitrates. Only compound **5** among the tested metabolites **1**–**5** has substantial FRS capacities. We take into consideration that compound **5** showed moderate vasodilation that was endothelial-dependent and inhibited by L-NAME but was not associated with NO generation. These data suggest preserving NO bioavailability rather than stimulating NO generation as the major mechanism of action of compound **5**. In the meantime, we cannot exclude the possibility that compound **5** may stimulate endothelium-derived hyperpolarizing factors or other vasodilators generated in the endothelium, such as prostacyclin. While compound **5** has not been previously investigated for its vasodilator effect, the xanthone group of compounds was reported to show vasorelaxant and antihypertensive activities [41]. While previous reports have shown the ability of flavonoids and benzophenone nuclei to enhance vasodilatation through NO production [35,42], our study is the first to introduce a similar effect for the phloroglucinol nucleus. Additionally, our study is the first to report the vasodilation activity of the xanthone nucleus through the inhibition of NO degradation. This finding is significant in terms of increased drug potency when NO production is intended. Interestingly, El-bassossy et al. [43] have reported a similar NO-protective mechanism by heme oxygenase-1. It is noteworthy to mention that the chloroform fraction of *H. revolutum* showed the highest potency relative to each isolated compound, which can be attributed to the synergistic effect of the bioactive compounds. The proposed pharmacological mechanism is illustrated in Figure 9.

The main limitation of this study is exploring vasodilation only in the thoracic aortic model; other arteries such as cerebral and abdominal arteries were not investigated. Additionally, the study concentrates only on NO from the endothelium as the main mechanism; other mechanisms such as Ca and K channels need to be investigated. Additionally, vasodilator activity was proven only in vitro; therefore, in vivo studies on animals are required, in detail, to assess the toxicity of these compounds as well as their metabolites and their effects on blood vessels. Finally, planning to assess the activity of the isolated compounds in humans should be a final step after the detailed study of these compounds.

## 5. Conclusions

The phytochemical investigation of the chloroform fraction of *H. revolutum* yielded two new compounds that were identified as revolutin (**1**) and hyperevolutin C (**2**), together with three known metabolites (**3**–**5**). Compounds **1**–**3** and **5** showed significant vasodilation in isolated aortae. The observed vasodilation of compounds **1**–**3** seems to be mediated via NO generation, as blocked by endothelial removal and L-NAME, and approves DAF-FM NO release. Compound **5** vasodilation is thought to be mediated by its free radical scavenging activities that protect the released NO from quenching by superoxides. Due to the multifactorial nature of cardiovascular diseases such as hypertension, knowing the mechanisms of the vasodilation action of these compounds is a crucial element for developing and planning different therapeutic strategies. Concretely, the observed vasodilation ability of these metabolites may reveal their potential therapeutic use against high-blood-pressure-related cardiovascular diseases.

## 6. Patents

This work resulted in US Patent number 10,780,139, 2020.

## Figures and Tables

**Figure 1:**
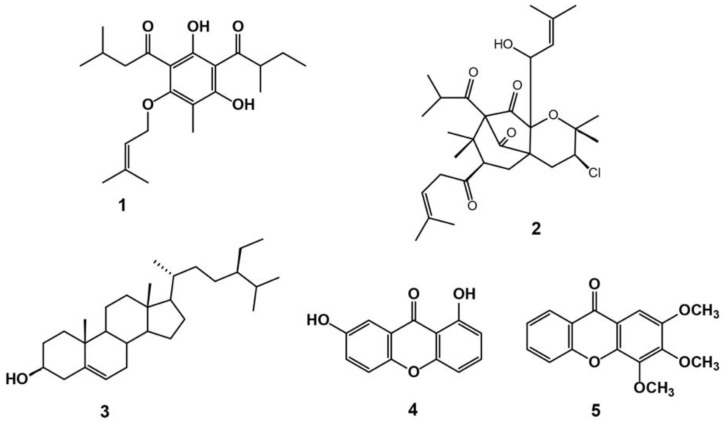
Chemical structures of isolated compounds **1**–**5**.

**Table 1:** NMR spectral data of compound **1** (CDCl_3_, 850 and 214 Hz).

No.	δ_H_ [Mult., *J* (Hz)]	δ_C_ (Mult.)	HMBC
1	-	162.4 C	
2	-	104.5 C	
3	-	149.9 C	
4	-	104.3 C	
5	-	162.5 C	
6	-	104.8 C	
7	4.52 d (6.0)	65.3 CH_2_	1, 8, 9, 10, 11
8	5.45 m	119.2 CH	7, 14, 15
9	-	138.3 C	-
10	1.79 s	25.8 CH_3_	8, 9, 11
11	1.73 s	18.3 CH_3_	8, 9, 10
12	-	205.7 C	-
13	2.94 d (6.0)	52.9 CH_2_	12, 14, 20, 21
14	1.65 m	25.3 CH	12, 15, 16
15	0.97 d (6.8)	22.8 CH_3_	13, 14
16	0.97 d (6.8)	22.8 CH_3_	13, 14
17	-	210.3 C	-
18	3.73 m	46.0 CH	4, 17, 19, 20, 21
19	1.84 m 1.41 m	26.9 CH_2_	18, 19, 20, 21
20	0.91 t (6.2)	12.0 CH_3_	18, 19
21	1.16 d (6.8)	16.7 CH_3_	17, 18, 19
22	2.02 s	7.3 CH_3_	1, 5, 6

**Table 2:** NMR spectral data of compound **2** (CDCl_3_, 850 and 214 Hz).

No.	δ_H_ [Mult., *J* (Hz)]	δ_C_ (Mult.)
2	-	83.0 C
3	2.40 dd (11.6, 8.3)	54.8 CH
4	2.12 dd (15.2, 11.6) 1.77 dd (15.2, 8.3)	25.6 CH_2_
4a	-	67.9 C
5	2.44 dd (15.1, 7.0) 1.92 d (15.0)	34.9 CH_2_
6	1.96 t (7.0)	42.4 CH
7	-	46.7 C
8	-	86.5 C
9	-	199.6 C
9a	-	76.9 C
10	-	202.4 C
11	1.38 s	31.1 CH_3_
12	1.26 s	25.3 CH_3_
13	1.28 s	22.2 CH_3_
14	1.24 s	24.6 CH_3_
15	-	207.0 C
16	1.78 m	44.3 CH
17	1.10 d (6.8)	21.1 CH_3_
18	1.09 d (6.8)	20.7 CH_3_
19	5.57 d (9.0)	74.7 CH
20	5.36 dt (9.0, 1.5)	119.8 CH
21	-	140.0 C
22	1.63 s	26.3 CH_3_
23	1.75 s	18.8 CH_3_
24	-	204.1 C
25	2.51 d (7.5)	28.7 CH_2_
26	5.22 brt (7.5)	118.7 CH
27	-	135.5 C
28	1.73 s	18.2 CH_3_
29	1.73 s	26.2 CH_3_

## Supplementary Materials

The following are available online at https://www.mdpi.com/article/10.3390/biology10060541/s1, Figure S1: HR-ESI, mass spectrometry spectrum of compound **1**., Figure S2: ^1^H NMR spectrum of compound **1** (CDCl_3_, 850 Hz), Figure S3: ^13^C NMR spectrum of compound **1** (CDCl_3_, 214 Hz), Figure S4: HSQC spectrum of compound **1**, Figure S5: HMBC spectrum of compound **1**, Figure S6: HR-ESI, mass spectrometry spectrum of compound **2**, Figure S7: ^1^H NMR spectrum of compound **2** (CDCl_3_, 850 Hz), Figure S8: ^13^C NMR spectrum of compound **2** (CDCl_3_, 214 Hz), Figure S9: HSQC spectrum of compound **2**, Figure S10: HMBC spectrum of compound **2**, Figure S11: COSY spectrum of compound **2**, Figure S12: NOESY spectrum of compound **2**, Figure S13: ^1^H NMR spectrum of compound **3** (CDCl_3_, 850 Hz), Figure S14: ^13^C NMR spectrum of compound **3** (CDCl_3_, 214 Hz), Figure S15: ^1^H NMR spectrum of compound **4** (euxanthone) (CDCl_3_, 850 Hz), Figure S16: ^13^C NMR spectrum of compound **4** (euxanthone) (CDCl_3_, 214 Hz), Figure S17: HSQC spectrum of compound **4** (euxanthone), Figure S18: HMBC spectrum of compound **4** (euxanthone), Figure S19: ^1^H NMR spectrum of compound **5** (2,3,4-tirmethoxy xanthone) (CDCl_3_, 850 Hz), Figure S20: ^13^C NMR spectrum of compound **5** (2,3,4-tirmethoxy xanthone) (CDCl_3_, 214 Hz), Figure S21: HSQC spectrum of compound **5** (2,3,4-tirmethoxy xanthone), Figure S22: HMBC spectrum of compound **5** (2,3,4-tirmethoxy xanthone), Table S1: NMR data of compound **3** (CDCl_3_, 850 and 214 Hz), Table S2: NMR data of compound **4** (euxanthone) (CDCl_3_, 850 and 214 Hz), Table S3: NMR data of compound **5** (2,3,4-tirmethoxy xanthone) (CDCl_3_, 850 and 214 Hz).
