# Supplementary material for: Nitric-Oxide-Mediated Vasodilation of Bioactive Compounds Isolated from Hypericum revolutum in Rat Aorta
Source: Biology (Basel). 2021 Jun 17;10(6):541. doi: 10.3390/biology10060541 (PMC8234642; doi:10.3390/biology10060541)
Supplement: Supplementary file 1 [file biology-10-00541-s001.zip › biology-1244635-supplementary.pdf]

Nitric oxide-mediated vasodilation of bioactive compounds isolated from *Hypericum revolutum*

. Abdallah[1,2,*], Noha Z.Hossam M

Timraz[1], Sabrin R. M. Ibrahim[3,4], Ali M El-Halawany[1,2], Azizah M. Malebari[5], Ibrahim A. Shehata[1,2], Hany M El-Bassossy[6,7]

Supplementary Figures

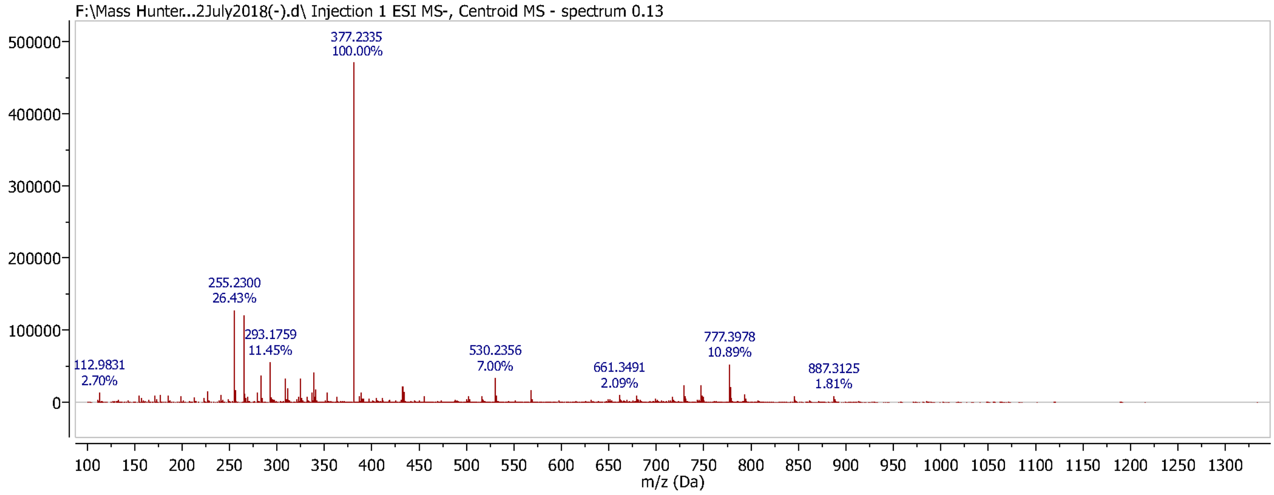

Supplementary Figure S1. HR- ESI, Mass spectrometry spectrum of compound **1.**

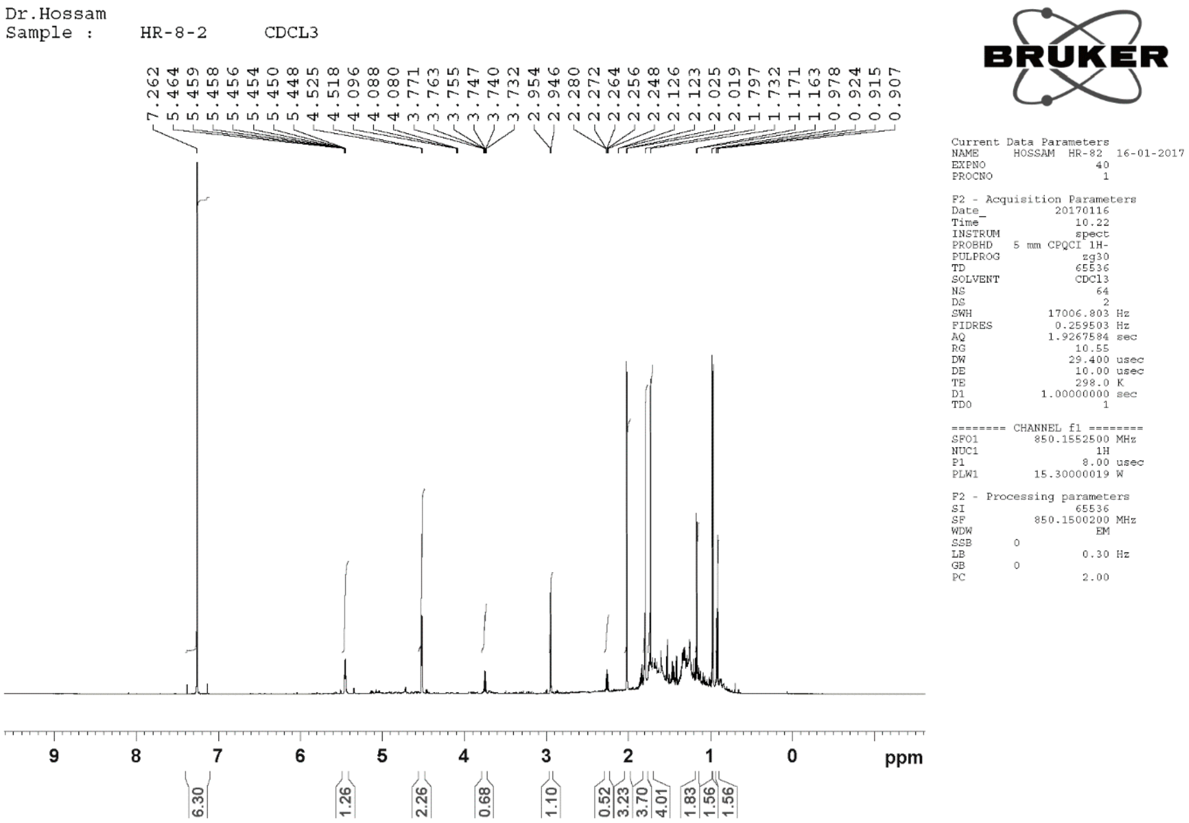

Supplementary Figure S2. ^{1}H NMR spectrum of compound **1** ($CDCl_3$, 850 Hz).

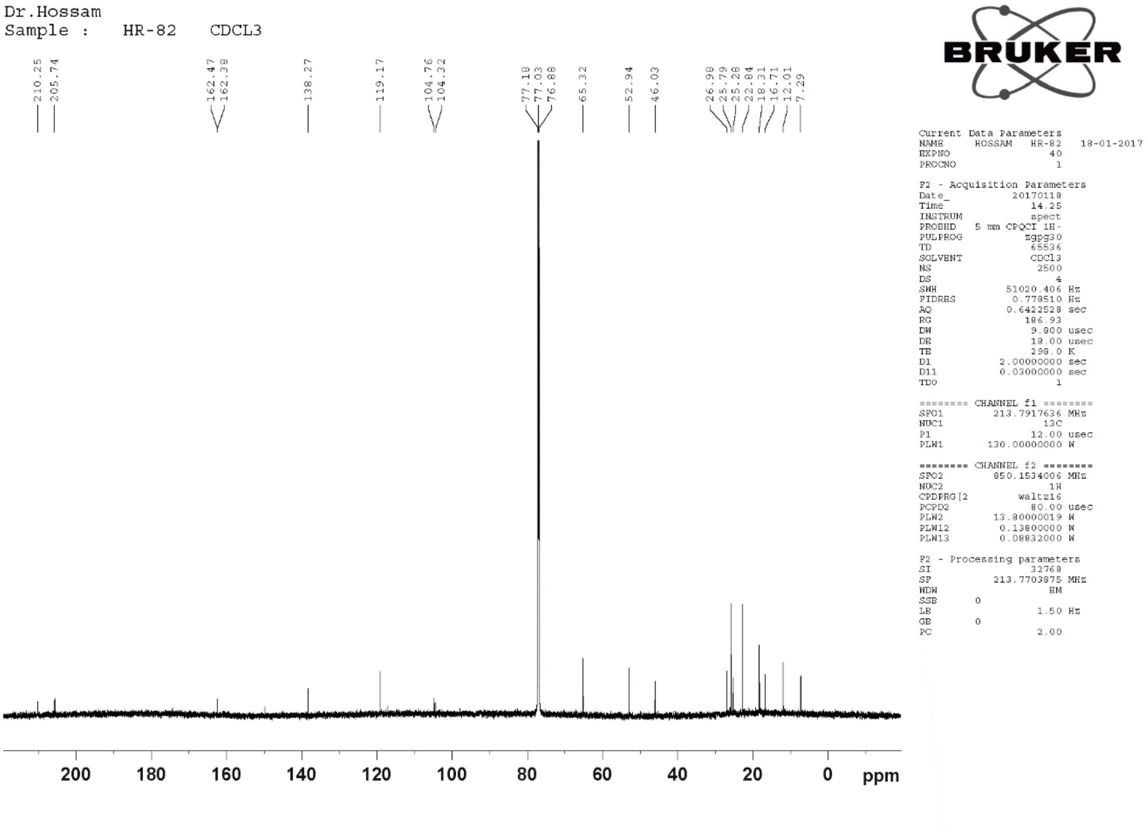

Supplementary Figure S3. ^{13}C NMR spectrum of compound **1** ($CDCl_3$, 214 Hz).

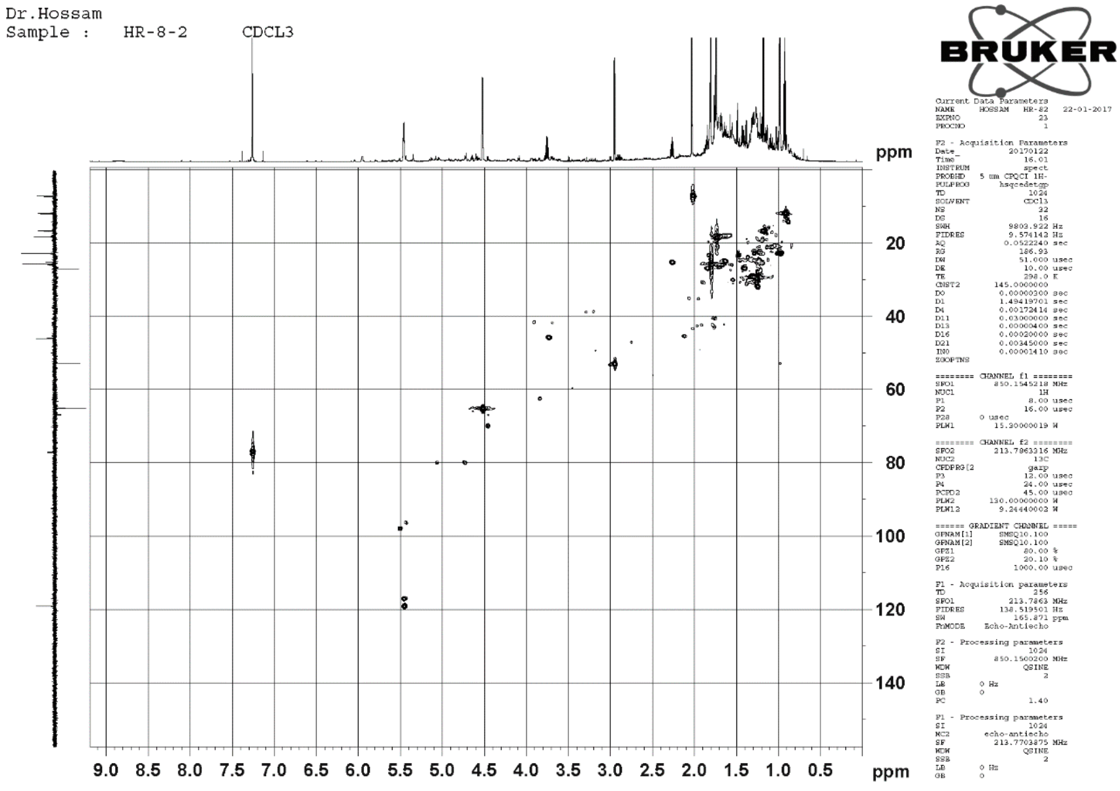

Supplementary Figure S4. HSQC spectrum of compound **1**

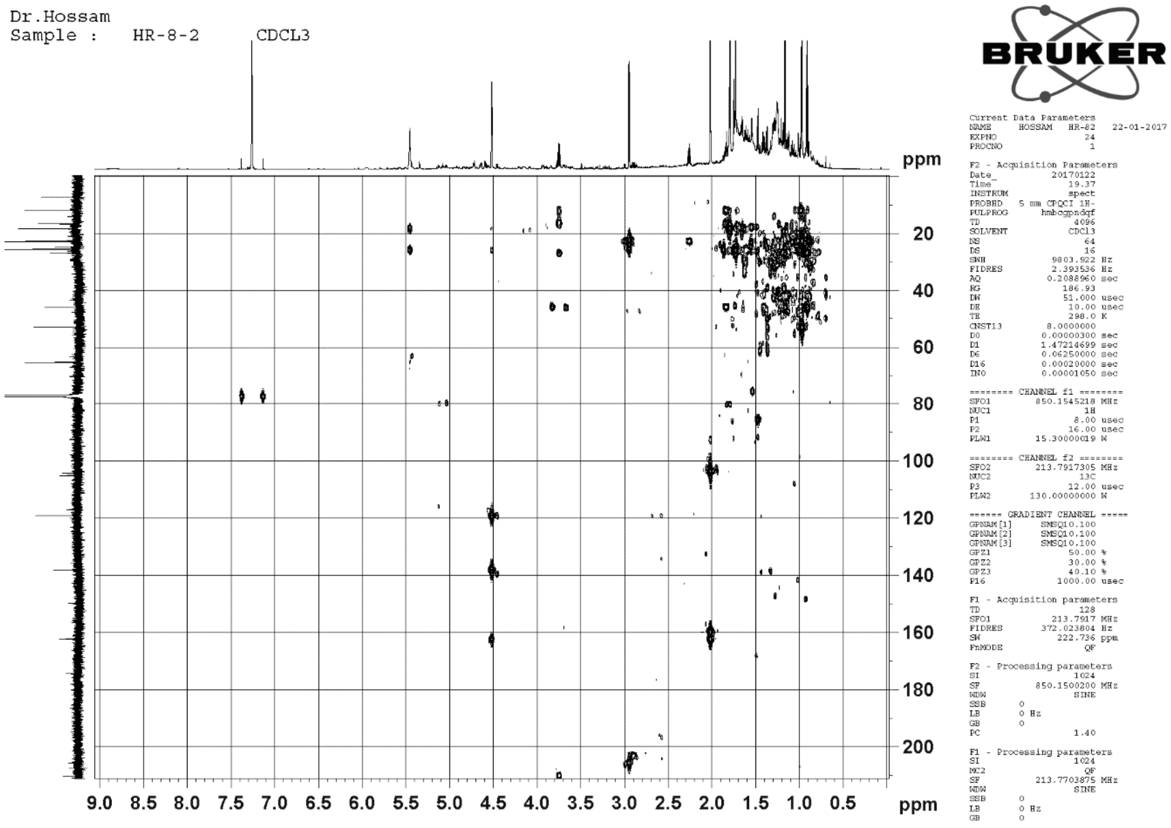

Supplementary Figure S5. HMBC spectrum of compound **1.**

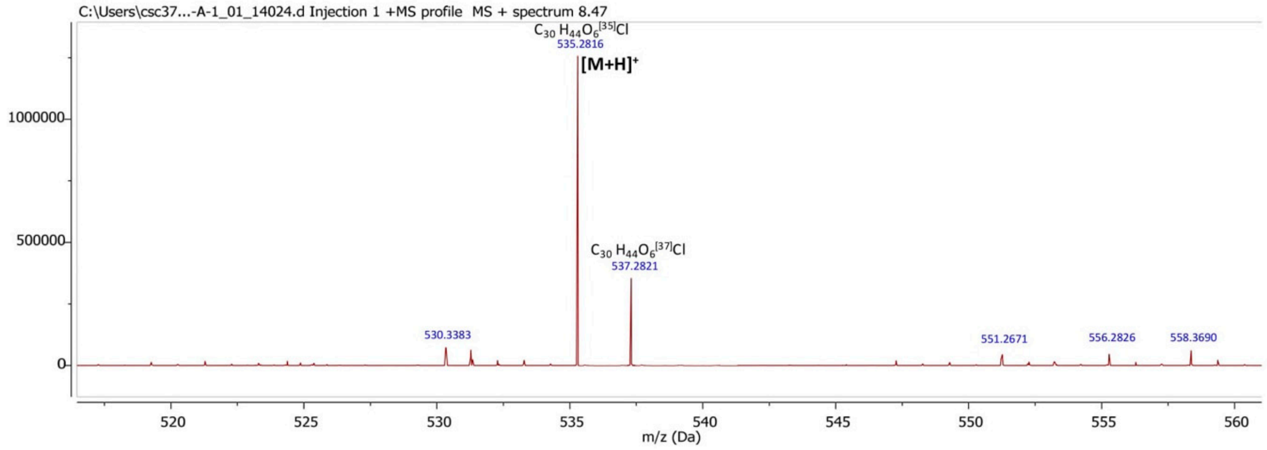

Supplementary Figure S6. HR- ESI, Mass spectrometry spectrum of compound **2.**

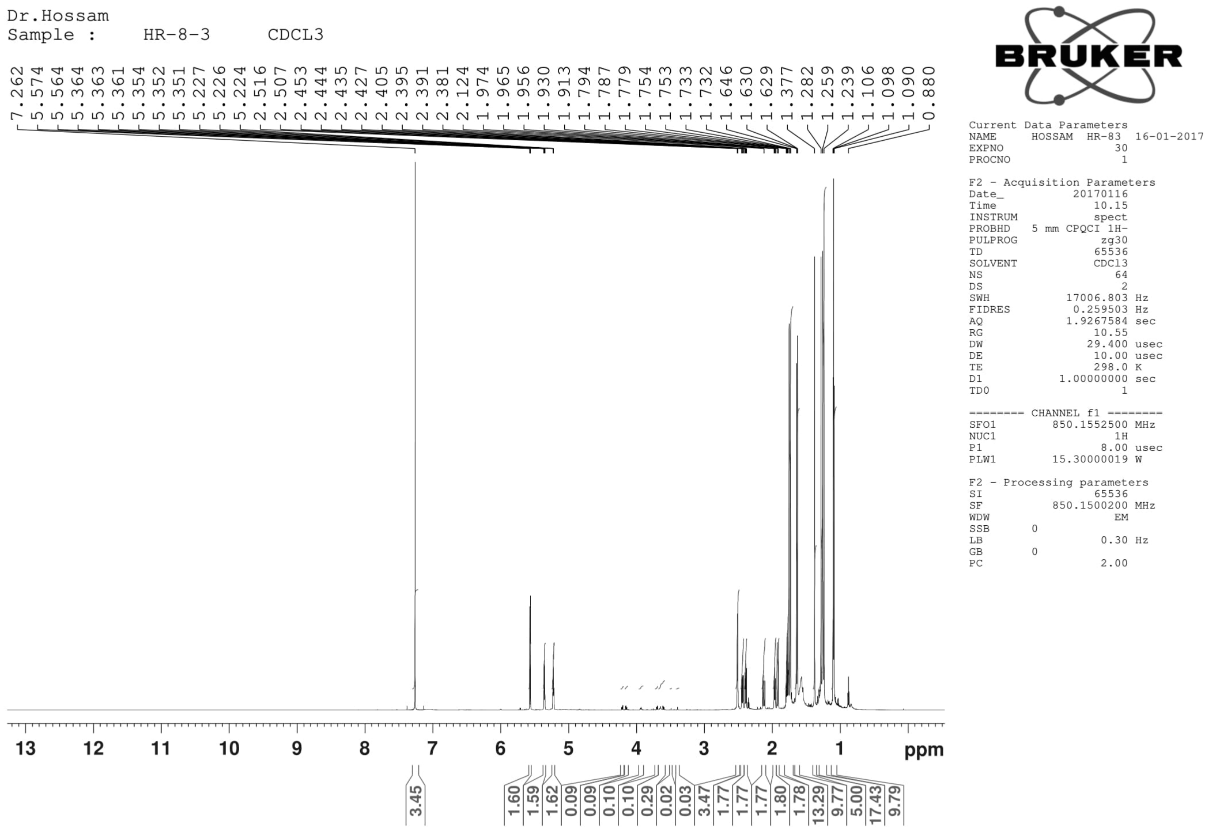

Supplementary Figure S7. ^{1}H NMR spectrum of compound **2** ($CDCl_3$, 850 Hz).

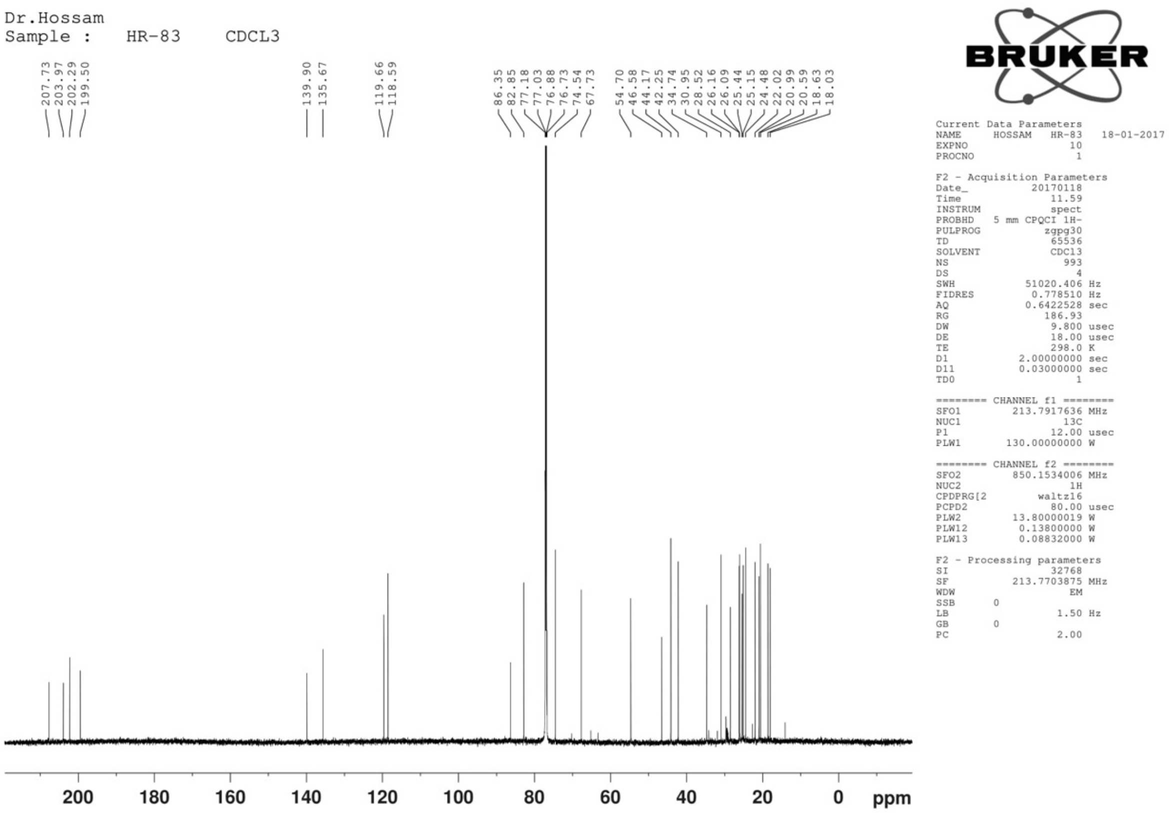

Supplementary Figure S8. ^{13}C NMR spectrum of compound **2** ($CDCl_3$, 214 Hz).

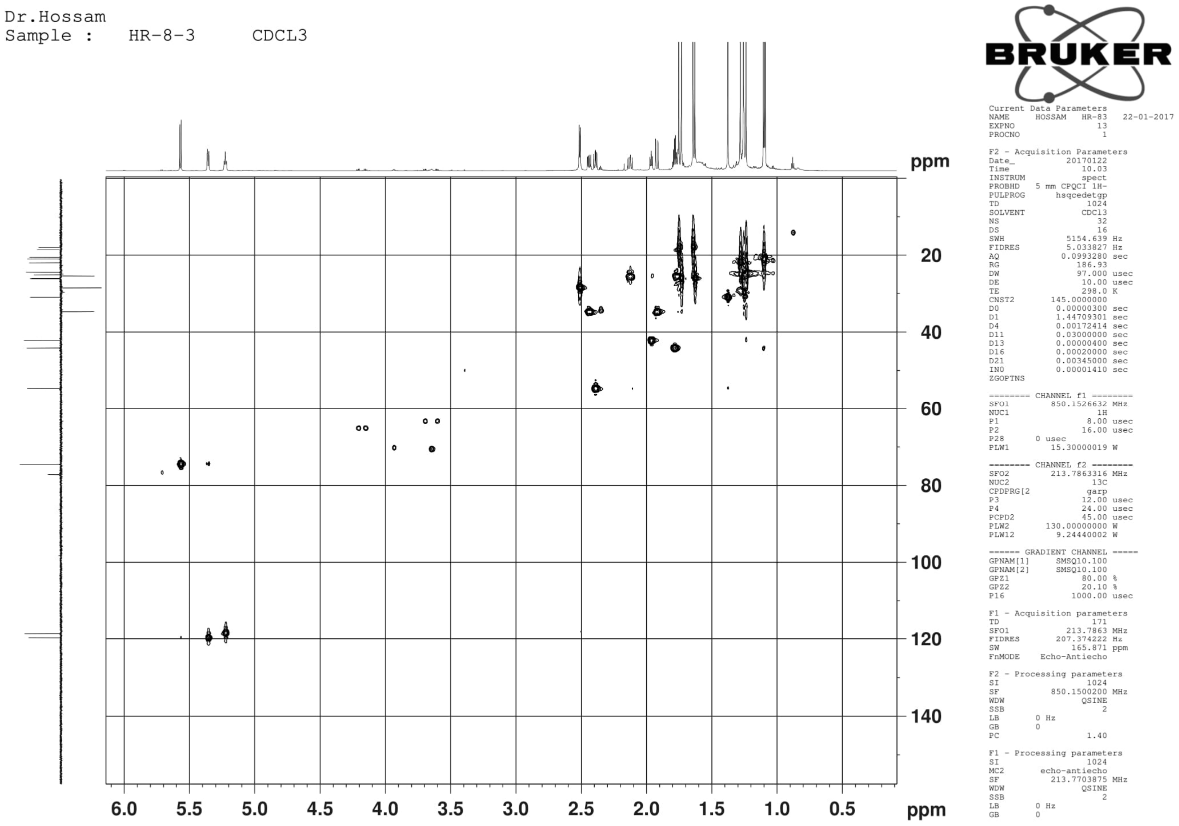

Supplementary Figure S9. HSQC spectrum of compound **2**

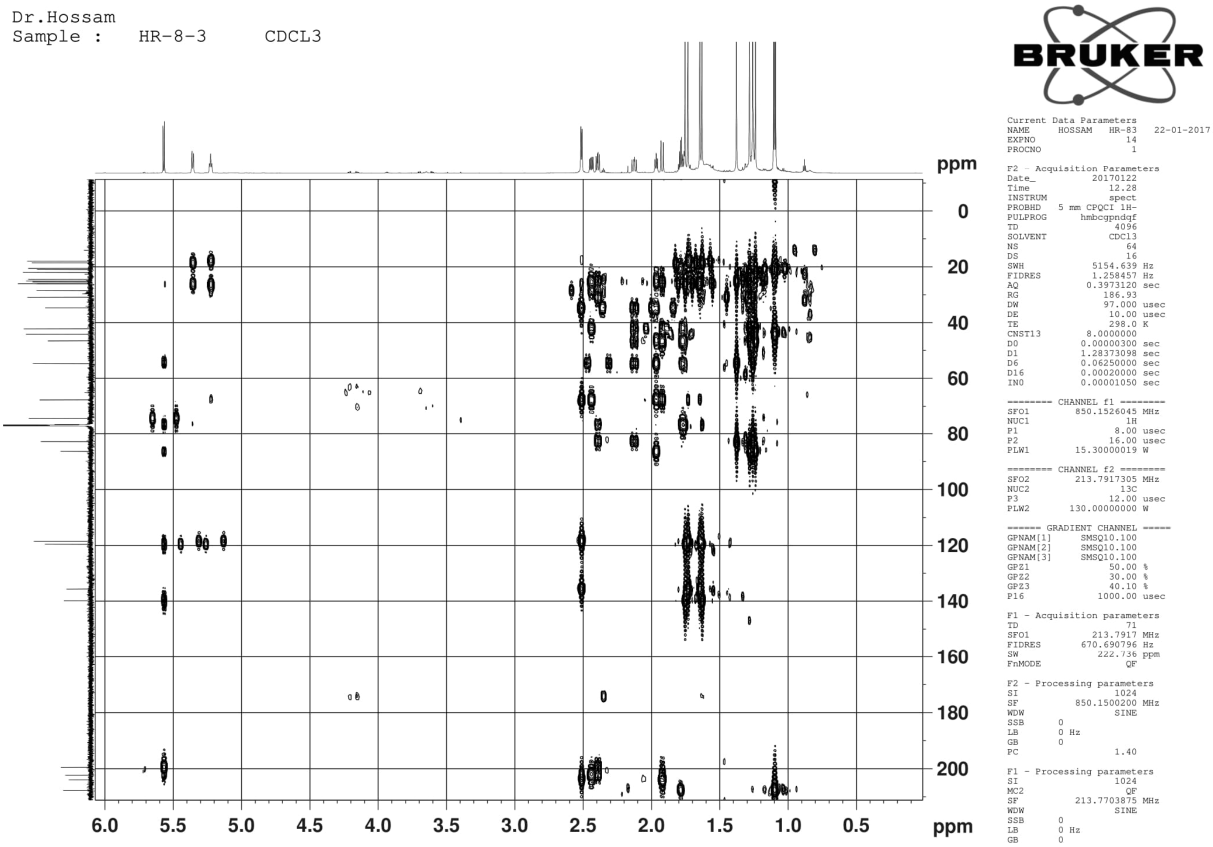

Supplementary Figure S10. HMBC spectrum of compound **2**

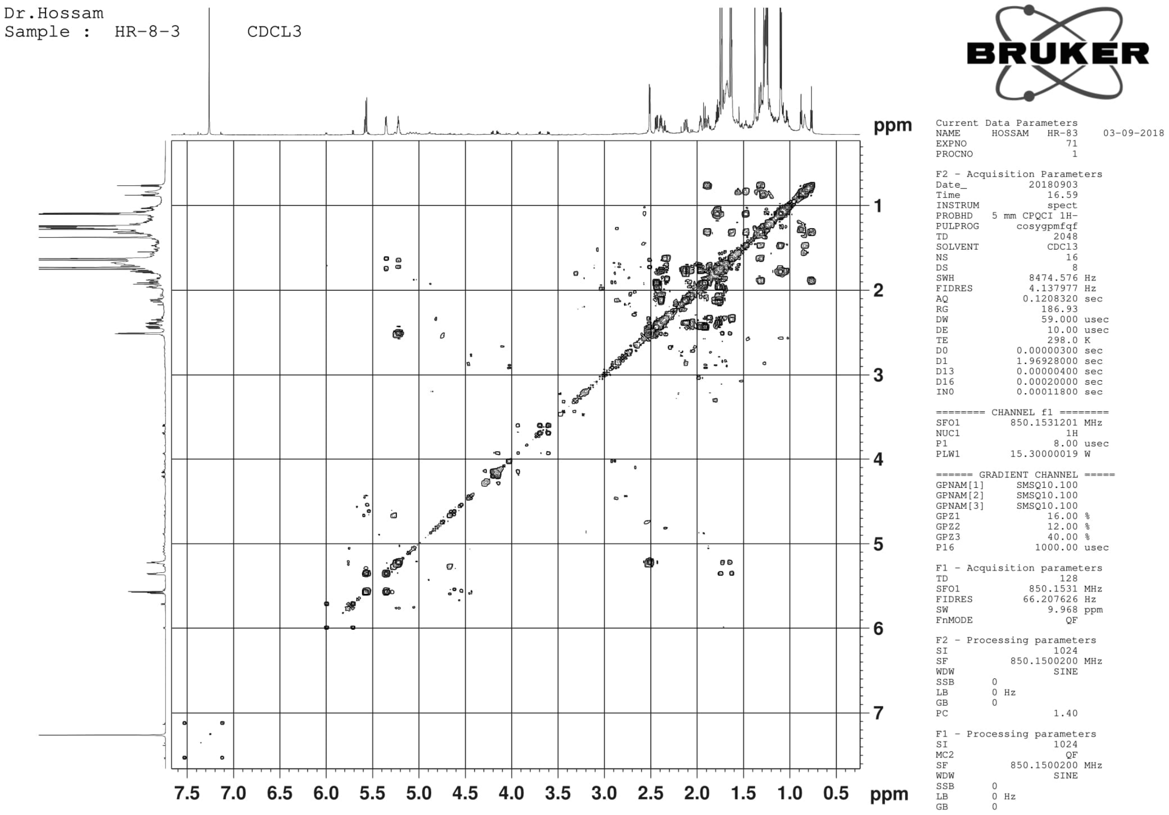

Supplementary Figure S11. COSY spectrum of compound **2**

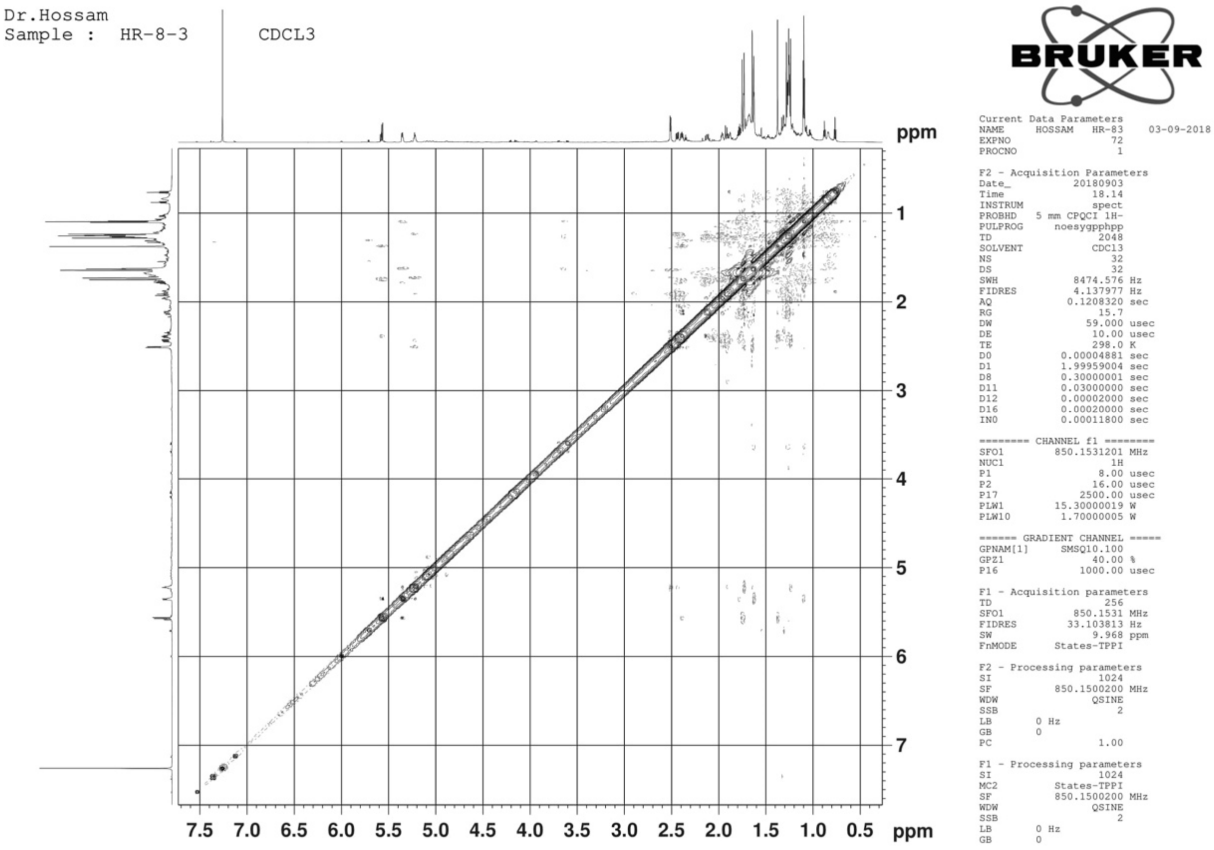

Supplementary Figure S12. NOESY spectrum of compound **2**

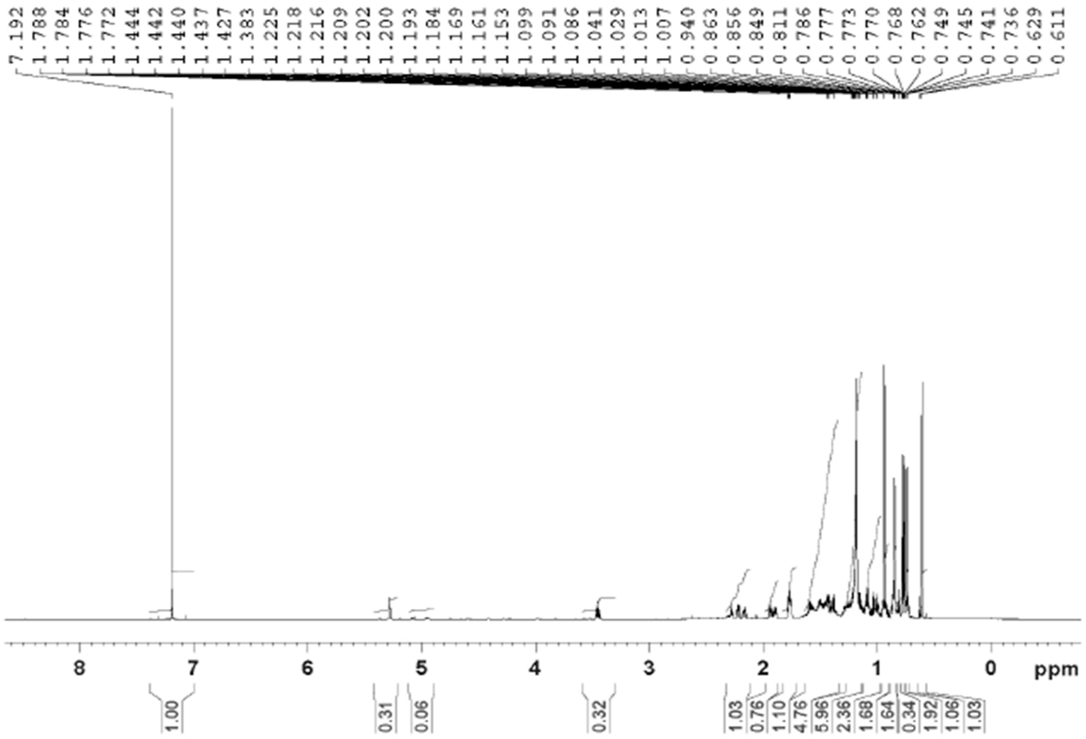

Supplementary Figure S13: ^{1}H NMR spectrum of compound **3** ($CDCl_3$, 850 Hz).

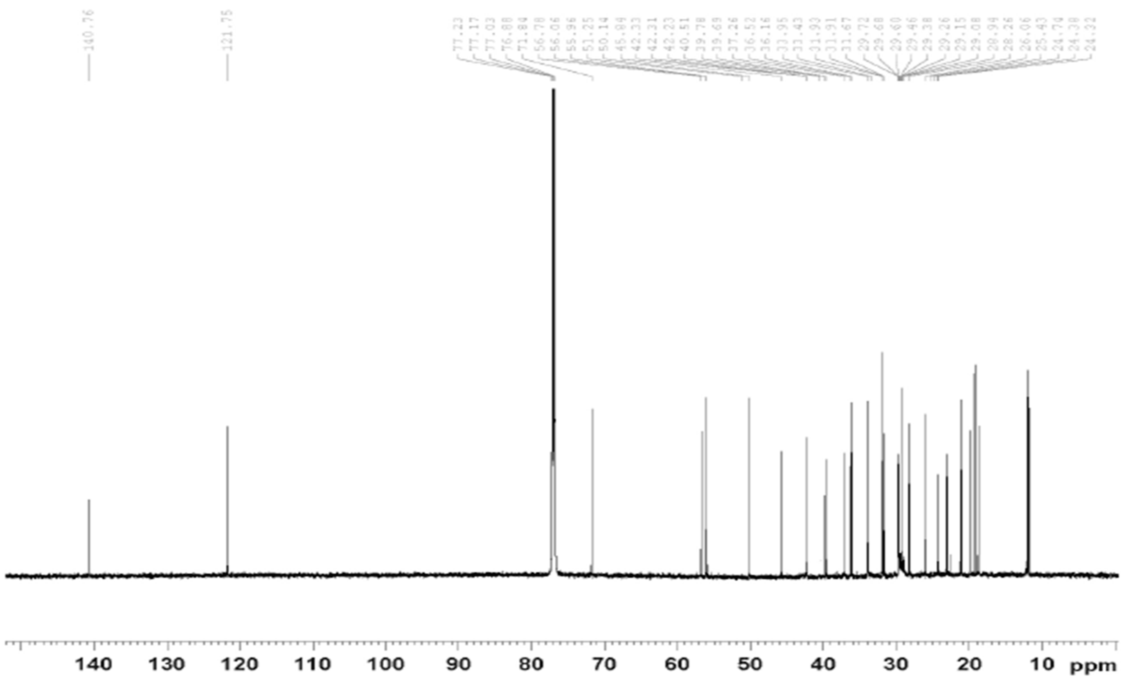

Supplementary Figure S14: ^{13}C NMR spectrum of compound **3** ($CDCl_3$, 214 Hz).

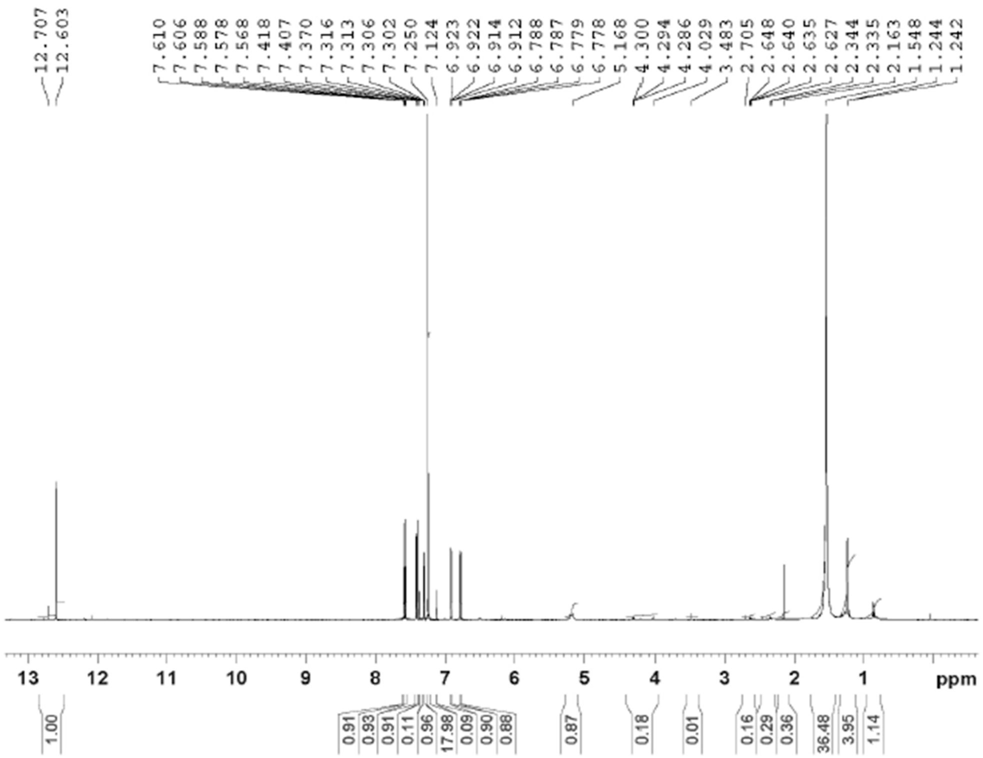

Supplementary Figure S15: ^{1}H NMR spectrum of compound **4** (euxanthone) ($CDCl_3$, 850 Hz).

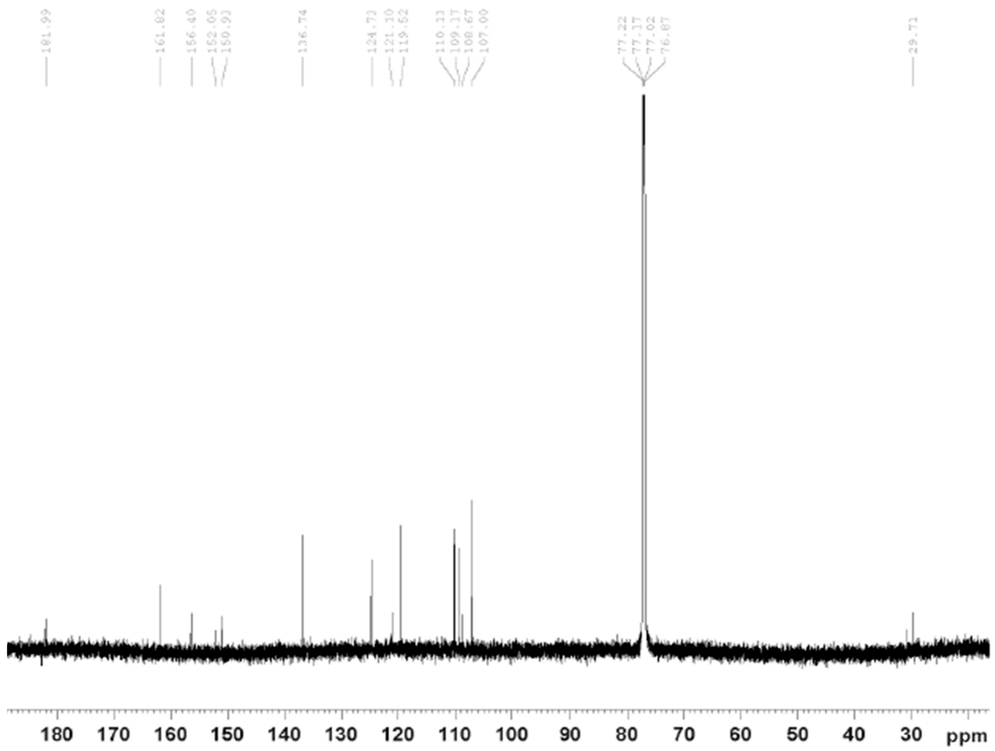

Supplementary Figure S16: ^{13}C NMR spectrum of compound **4** (euxanthone) ($CDCl_3$, 214 Hz).

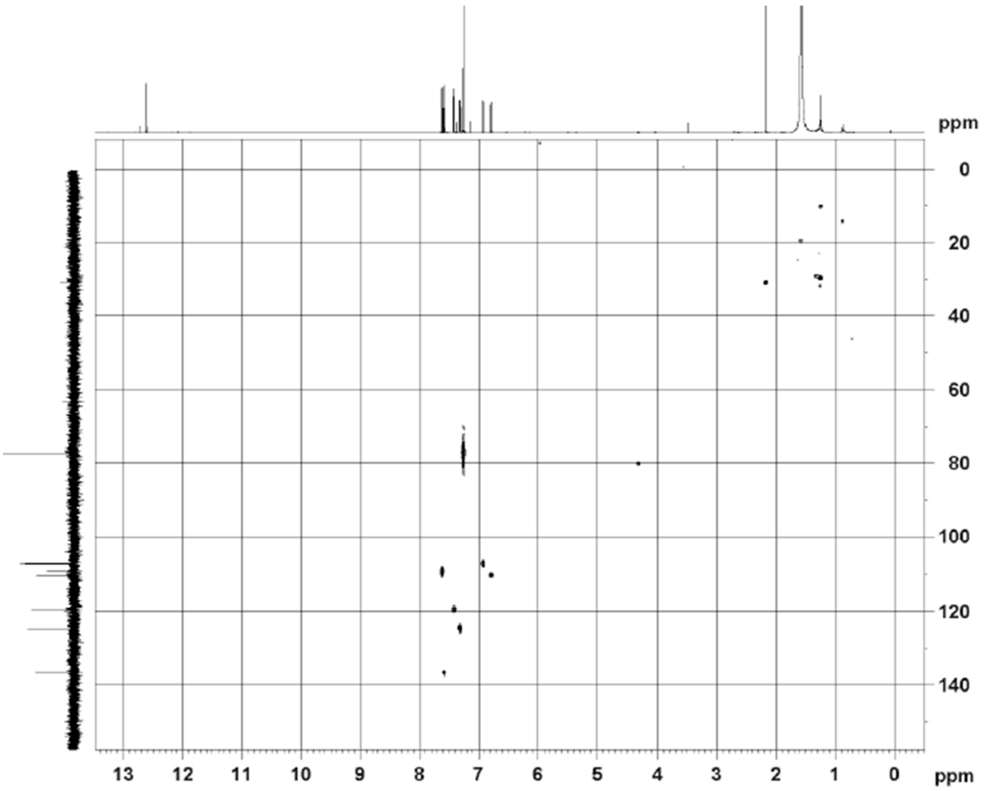

Supplementary Figure S17: HSQC spectrum of compound **4** (euxanthone)

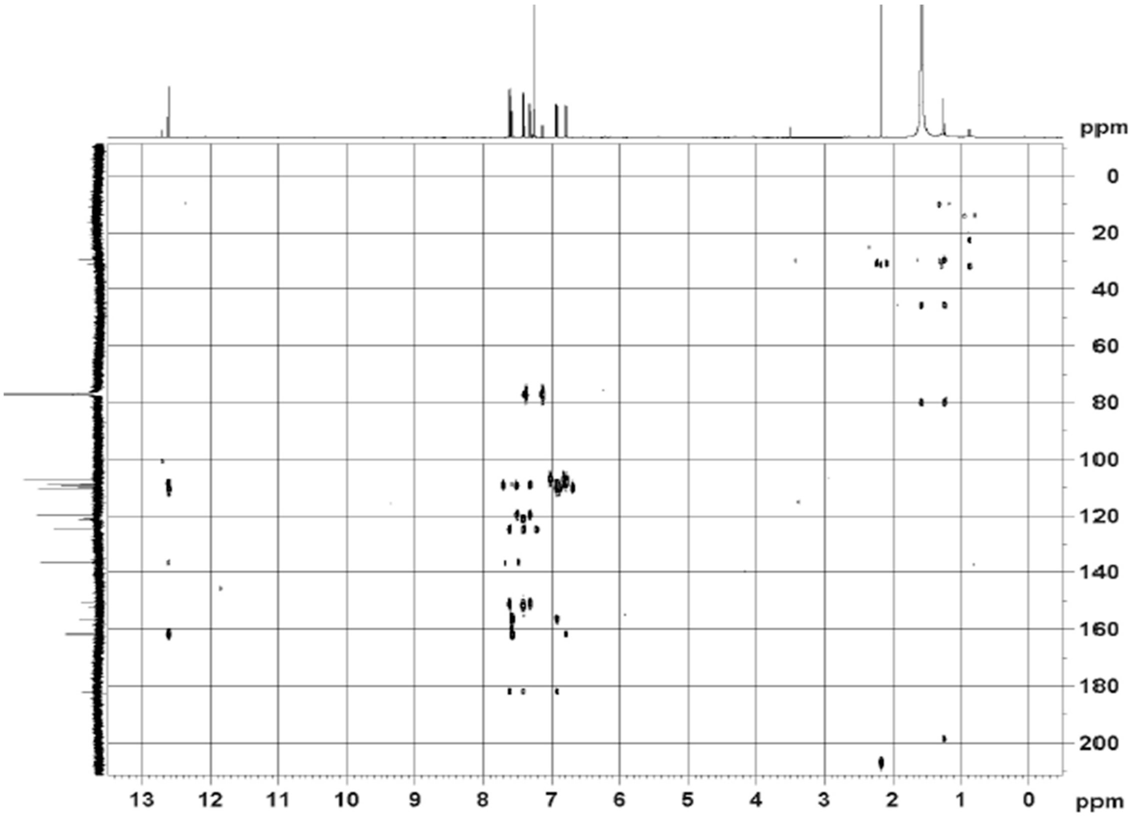

Supplementary Figure S18: HMBC spectrum of compound **4** (euxanthone)

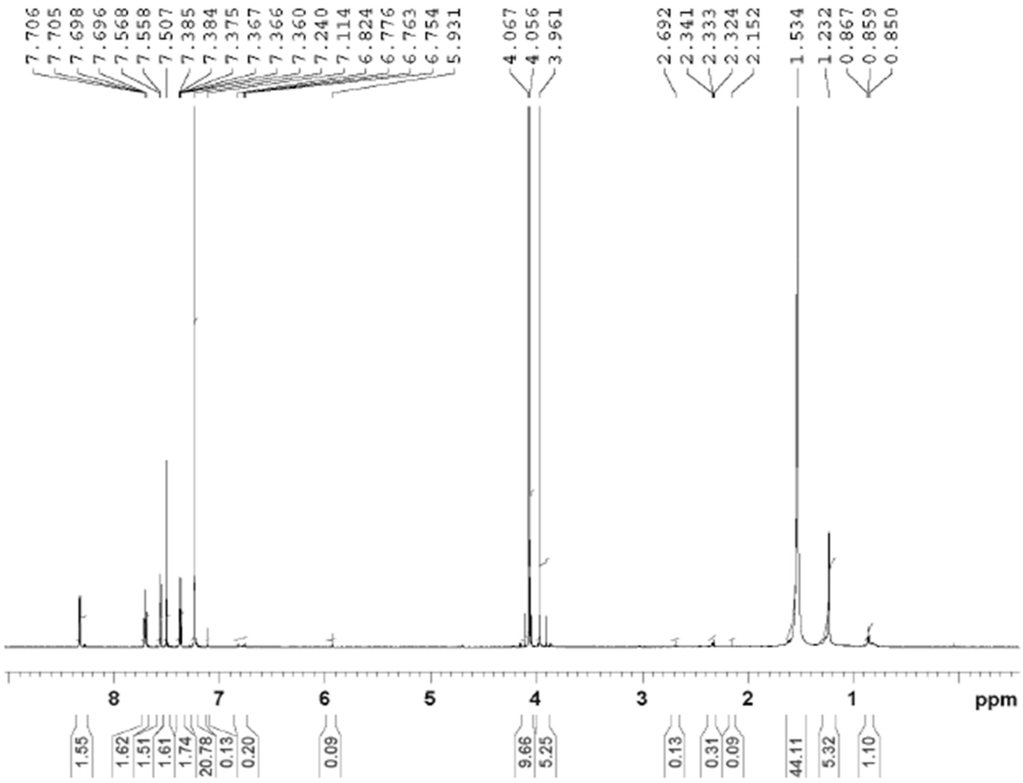

Supplementary Figure S19: ^{1}H NMR spectrum of compound **5** (2,3,4-tirmethoxy xanthone) ($CDCl_3$, 850 Hz).

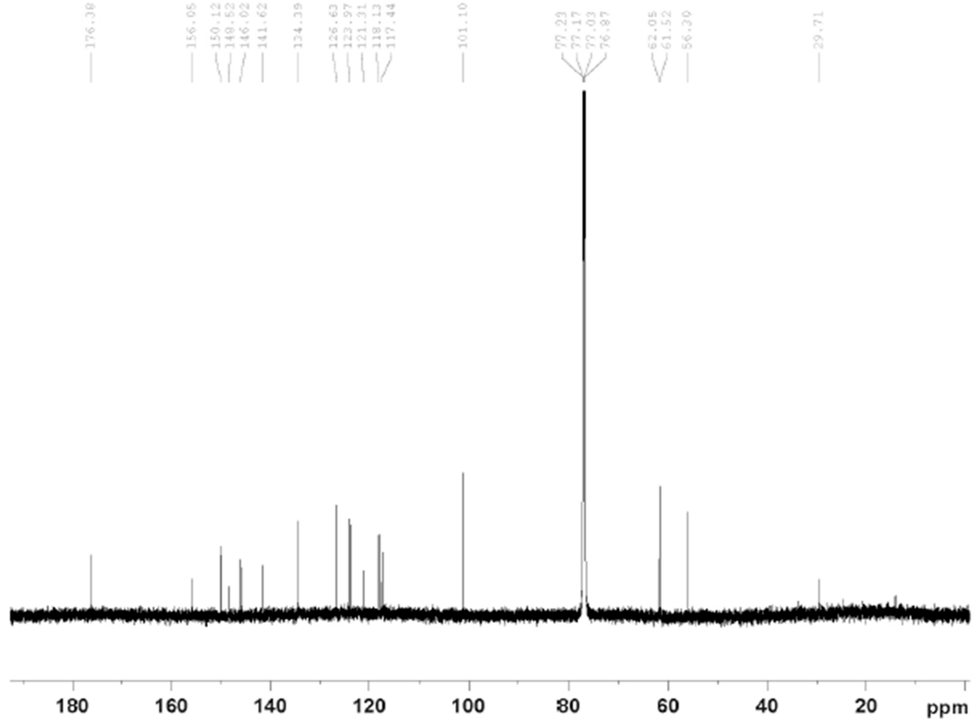

Supplementary Figure S20: ^{13}C NMR spectrum of compound **5** (2,3,4-tirmethoxy xanthone) ($CDCl_3$, 214 Hz).

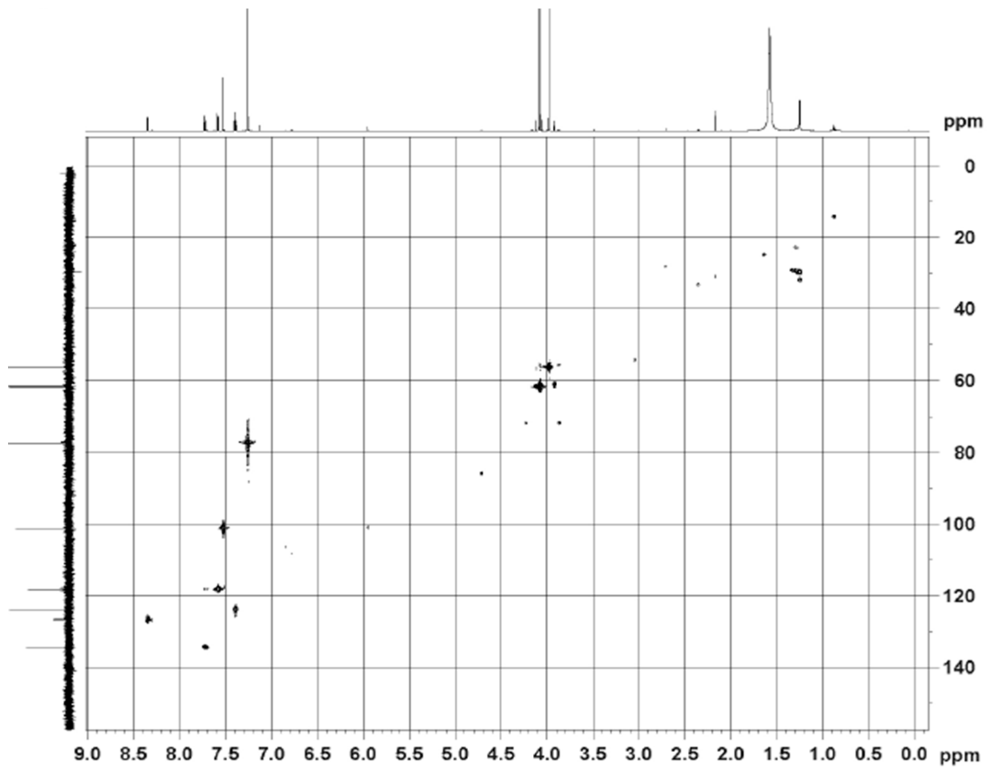

Supplementary Figure S21: HSQC spectrum of compound **5** (2,3,4-tirmethoxy xanthone)

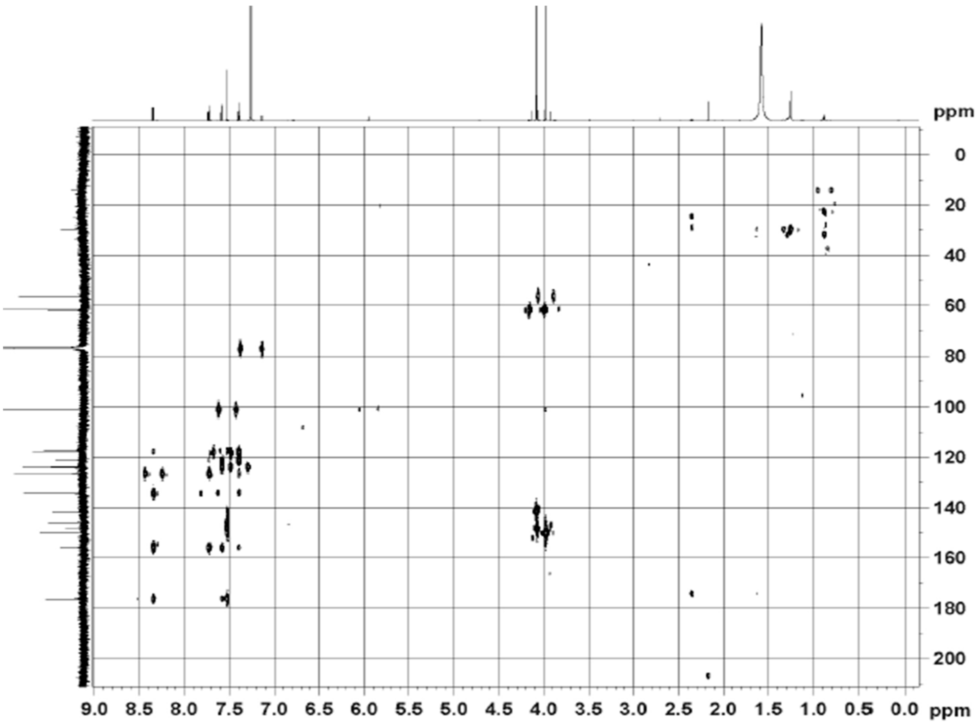

Supplementary Figure S22: HMBC spectrum of compound **5** (2,3,4-tirmethoxy xanthone)

Supplementary Tables

Supplementary Table S1: NMR data of compound **3** ($CDCl_3$, 850 and 214 Hz).

No.	δ_H [mult., *J* (Hz)]	δ_C (mult.)
**1**	1.83, 1.07 m	37.3 CH_2
**2**	1.45, 1.23 m	31.9 CH_2
**3**	3.45 m	71.8 CH
**4**	1.95,1.83 m	42.3 CH_2
**5**	-	140.8 C
**6**	5.28 m	121.8 CH
**7**	1.97, 1.83 m	31.7 CH_2
**8**	1.51 m	31.9 CH
**9**	0.92 m	50.1CH
**10**	-	36.5 C
**11**	1.45 m	21.1 CH_2
**12**	1.99 ,1.51 m	39.7 CH_2
**13**	2.28, 2.23 m	42.3 CH_2
**14**	0.98 m	56.8CH
**15**	1.58, 1.03 m	24.3 CH_2
**16**	1.84, 1.25 m	28.3 CH_2
**17**	1.09 m	56.1 CH
**18**	0.61 s	11.9 CH_3
**19**	0.94 s	19.4 CH_3
**20**	1.38 m	36.2 CH
**21**	0.84 d (6.4)	19.0 CH_3
**22**	1.32, 1.11 m	34.0 CH_2
**23**	1.13 m	26.1 CH_2
**24**	0.93 m	45.8 CH
**25**	1.65 m	29.2 CH
**26**	0.74 d (6.7)	18.8 CH_3
**27**	0.76 d (6.7)	19.8 CH_3
**28**	-	23.1 CH_2
**29**	0.77 t (6.9)	12.0 CH_3

Supplementary Table S2: NMR data of compound **4** (euxanthone) ($CDCl_3$, 850 and 214 Hz).

**No.**	**δ_H [mult., *J* (Hz)]**	**δ_C (mult.)**	**HMBC**
**1**	-	161.8 C	-
**2**	6.78 dd (2.1, 8.5)	110.1 CH	1, 2, 8b
**3**	7.58 t (8.5)	136.7 CH	1, 4a, 8b
**4**	6.92 dd (2.1, 8.5)	107.0 CH	2, 4a, 8b
**4a**	-	156.4 C	-
**4b**	-	150.9 C	-
**5**	7.41 d (8.5)	119.5 CH	6, 7, 8a
**6**	7.31 dd (2.5, 8.5)	124.7 CH	5, 8, 4b
**7**	-	152.1 C	-
**8**	7.61 d (2.5)	109.2 CH	-
**8a**	-	121.1C	-
**8b**	-	108.7 C	-
**9**	-	182.0 C	
**1-OH**	12.6 s	-	1, 3, 4a, 8b

Supplementary Table S3: NMR data of compound **5** (2,3,4-tirmethoxy xanthone) ($CDCl_3$, 850 and 214 Hz).

**No.**	**δ_H [mult., *J* (Hz)]**	**δ_C (mult.)**	**HMBC**
1	7.51 s	101.1 CH	3, 4a, 9
2	-	141.6 C	-
3	-	148.5 C	-
4	-	150.1 C	-
4a	-	146 .2 C	-
4b	-	156.1 C	-
5	7.56 brd (8.5)	118.1 CH	7, 8a
6	7.71 dt (1.7, 8.5)	134.4 CH	8, 4b
7	7.38 t (8.5)	124.0 CH	3, 5, 8a
8	8.32 dd (1.7, 8.5)	126.6 CH	4b, 6
8a	-	121.1Cs	-
8b	-	117.4 C	-
9	-	176.4 C	-
2-OCH_3	4.07 s	56.3 CH_3	2, 8b
3-OCH_3	4.06 s	61.5 CH_3	3, 4a
4-OCH_3	3.96 s	61.1 CH_3	4, 8b
